# An Injectable BMP‐2‐Releasing Porous Hydrogel Regulating the Paracrine Effects of ADSCs Promotes Tendon‐to‐Bone Healing in Rotator Cuff Repair

**DOI:** 10.1002/advs.202415923

**Published:** 2025-06-23

**Authors:** Meijuan Yuan, Xiaomei Dai, Yuxia Yang, Shuai Liu, Ziyi Xu, Liang Wang, Junli Shi, Wenkang Liu, Jian Yang, Dianwei Liu, Hang Yao, Wenyong Fei

**Affiliations:** ^1^ School of Nursing and School of Public Health Yangzhou University Yangzhou 225001 P. R. China; ^2^ Center of Basic and Clinical Research in Sports Medicine Yangzhou University Yangzhou Jiangsu 225001 P. R. China; ^3^ Department of Orthopedics and Sports Medicine Northern Jiangsu People's Hospital Affiliated to Yangzhou University Yangzhou 225001 P. R. China; ^4^ Medical College Yangzhou University Yangzhou 225001 P. R. China; ^5^ Department of Orthopedics and Sports Medicine The Yangzhou school of clinical medicine of Dalian Medical University Dalian 116044 P. R. China; ^6^ School of Chemistry and Chemical Engineering Yangzhou University Yangzhou 225009 P. R. China

**Keywords:** ADSCs, BMP‐2, paracrine effects, porous hydrogels, tendon‐to‐bone healing

## Abstract

Rotator cuff tears often require surgical reconstruction; however, outcomes remain suboptimal, primarily because of the challenges in fully restoring the tendon–bone interface (TBI). The normal fibrocartilaginous transition zone is often replaced by fibrous scar tissue, thereby increasing the risk of retears. A porous hyaluronic acid methacrylate hydrogel is developed to encapsulate adipose‐derived stem cells (ADSCs) and bone morphogenetic protein‐2 (BMP‐2). Gelatin microspheres served as porogens to create micropores within the hyaluronic acid methacrylate hydrogel (HMs), and hollow gelatin methacryloyl hydrogel microspheres are used to encapsulate BMP‐2 (GMBs). In vitro experiments confirm substantial proliferation and homing of ADSCs within the porous hydrogel system. When the ADSCs‐loaded composite hydrogel (HMs/MBs/ADSCs) is applied to a rat rotator cuff injury model, it effectively reconstructs the fibrocartilage transition zone and promotes TBI healing. Transcriptome sequencing is performed to elucidate the mechanisms underlying BMP‐2‐induced fibrocartilage reconstruction. Furthermore, integrated in vitro sequencing reveals that HMs/MBs/ADSCs modulated the tissue microenvironment to enhance tissue regeneration, primarily through BMP‐2′s regulation of the paracrine effects of ADSCs. This study explores the comprehensive pathway through which HMs/MBs/ADSCs enhance tissue regeneration, emphasizing the synergistic effects of BMP‐2 and ADSCs, and offers novel insights into the mechanisms underlying TBI healing.

## Graphical abstract

This study designs an injectable porous hydrogel (HMs/MBs/ADSCs) integrating hollow microspheres for sustained BMP‐2 release to enhance adipose‐derived stem cells' paracrine effect. It accelerates tendon‐bone healing in rat rotator cuff injuries via spatiotemporal regulation: early AMPK‐mediated mitochondrial protection and late TGF‐β/Smad2/3‐driven fibrocartilage regeneration. By integrating material design and mechanistic exploration, this study enhances the application potential of tissue‐engineered hydrogels.

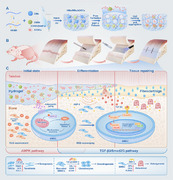

## 1 Introduction

Rotator cuff tears cause shoulder pain and limited mobility and frequently necessitate surgical repair, with outcomes gradually improving as medical technology advances.^[^ ^1^ ^]^ However, 20–70% of patients continue to experience postoperative functional limitations and retears.^[^ ^2^ ^]^ The critical site of failure is the tendon–bone interface (TBI), which in its natural state consists of a four‐layer gradient structure composed of tendon, uncalcified fibrocartilage, calcified fibrocartilage, and bone.^[^ ^3^ ^]^ Surgically reconstructed TBI develops fibrovascular scar tissue composed primarily of type III collagen, and its limited mechanical strength leads to a high rate of retears.^[^ ^4^ ^]^ Furthermore, the number of cells at the interface gradually diminishes, and organized collagen fibers fail to form, thereby preventing restoration of the characteristic fibrocartilage transition zone.^[^ ^5^ ^]^ Achieving effective integration in patients with TBI remains challenging. Therefore, researchers have explored various tissue engineering strategies to enhance rotator cuff healing.^[^ ^6^ ^]^

The complex native structure of the TBI makes it difficult to establish a suitable biochemical microenvironment without external intervention after the interface is damaged. Therefore, the design of appropriate microenvironmental guidance materials is essential for facilitating regenerative cell migration, proliferation, and signal transduction.^[^ ^7^ ^]^ Cell interactions on flat biomaterial surfaces have commonly been studied in vitro.^[^ ^8^ ^]^ However, cells attached to 2D structures do not realistically reproduce the extracellular matrix (ECM) environment.^[^ ^9^ ^]^ To address these limitations and accommodate defects with irregular shapes, injectable biomaterials have attracted considerable attention. Hydrogels have been widely used because their prepolymeric forms closely mimic the physicochemical properties of ECM. Hydrogels offer a 3D platform for cell encapsulation and the incorporation and release of bioactive ingredients.^[^ ^10^ ^]^ Additionally, the influence of hydrogels on cell behavior is multifaceted, and optimizing their internal structure can enhance cell compatibility, tissue integration, and host responses.^[^ ^11^ ^]^ Among these, increasing porosity or pore size often enhances ECM secretion, cell infiltration, tissue growth, and molecular delivery. Because of the size limitations of hydrogels, researchers have developed several methods for creating macroporous hydrogels, including gas foaming, freeze‐drying, and particle leaching.^[^ ^12^ ^]^ However, most methods require cell seeding after hydrogel formation, which hinders uniform 3D cell encapsulation owing to challenges in controlling cell distribution. Therefore, it is necessary to use a cost‐effective and highly biocompatible porogen to construct porous hydrogel systems that provide a more suitable 3D microenvironment for cells.

From the tissue microenvironment perspective, rotator cuff injuries are often accompanied by microenvironmental damage, resulting in excessive production of reactive oxygen species (ROS).^[^ ^13^ ^]^ Concurrently, the prolonged accumulation of ROS increases the secretion of proinflammatory cytokines, thereby maintaining a chronic inflammatory cycle.^[^ ^14^ ^]^ Stem cells and bioactive factors are widely acknowledged as crucial for inhibiting oxidative stress and reducing ROS production.^[^ ^15^ ^]^ The therapeutic effects of stem cells rely primarily on their paracrine functions. Paracrine factors or exosomes derived from stem cells can migrate to inflamed sites and regulate the activity and function of target cells via intracellular communication.^[^ ^16^ ^]^ The biological activity of stem cells can downregulate the NF‐κB pathway, reduce inflammation, and promote TBI healing.^[^ ^17^ ^]^ Among these, adipose‐derived stem cells (ADSCs), which are known for their accessibility and potent paracrine effects, have become a key focus of regenerative therapies.^[^ ^18^ ^]^ Therefore, using ADSCs as seed cells may enhance the biological functionality of tissue‐engineered hydrogels.

In addition to pathogen clearance and inflammation control, the integration of growth factors is crucial for promoting tissue repair. Growth factors are essential regulators of cellular processes. They regulate the paracrine activity of exogenous stem cells and manipulate endogenous stem cells to activate the inherent natural repair mechanisms.^[^ ^19^ ^]^ Various growth factors have been investigated for TBI, such as platelet‐derived growth factor, vascular endothelial growth factor, and bone morphogenetic protein. However, most growth factors are unsuitable for long‐term tissue repair.^[^ ^20^ ^]^ Bone morphogenetic protein 2 (BMP‐2) has been used clinically because of its strong bone‐inducing capability.^[^ ^21^ ^]^ BMP‐2 induces chondrogenic differentiation and enhances the synthesis of fibrous cartilage ECM components, proteoglycans, and type II collagen.^[^ ^22^ ^]^ Therefore, BMP‐2 may serve as a key signaling molecule for enhancing the paracrine activity of stem cells and reconstructing the fibrous cartilage transition zone. Numerous studies have reported the positive effects of BMP‐2 on TBI healing and clarified the related mechanisms.^[^ ^23^ ^]^ However, no studies have elucidated the mechanism by which BMP‐2 coordinates with stem cells throughout the transition from initial trauma to later repair, which may represent a novel breakthrough in TBI healing.

**Figure 1:**
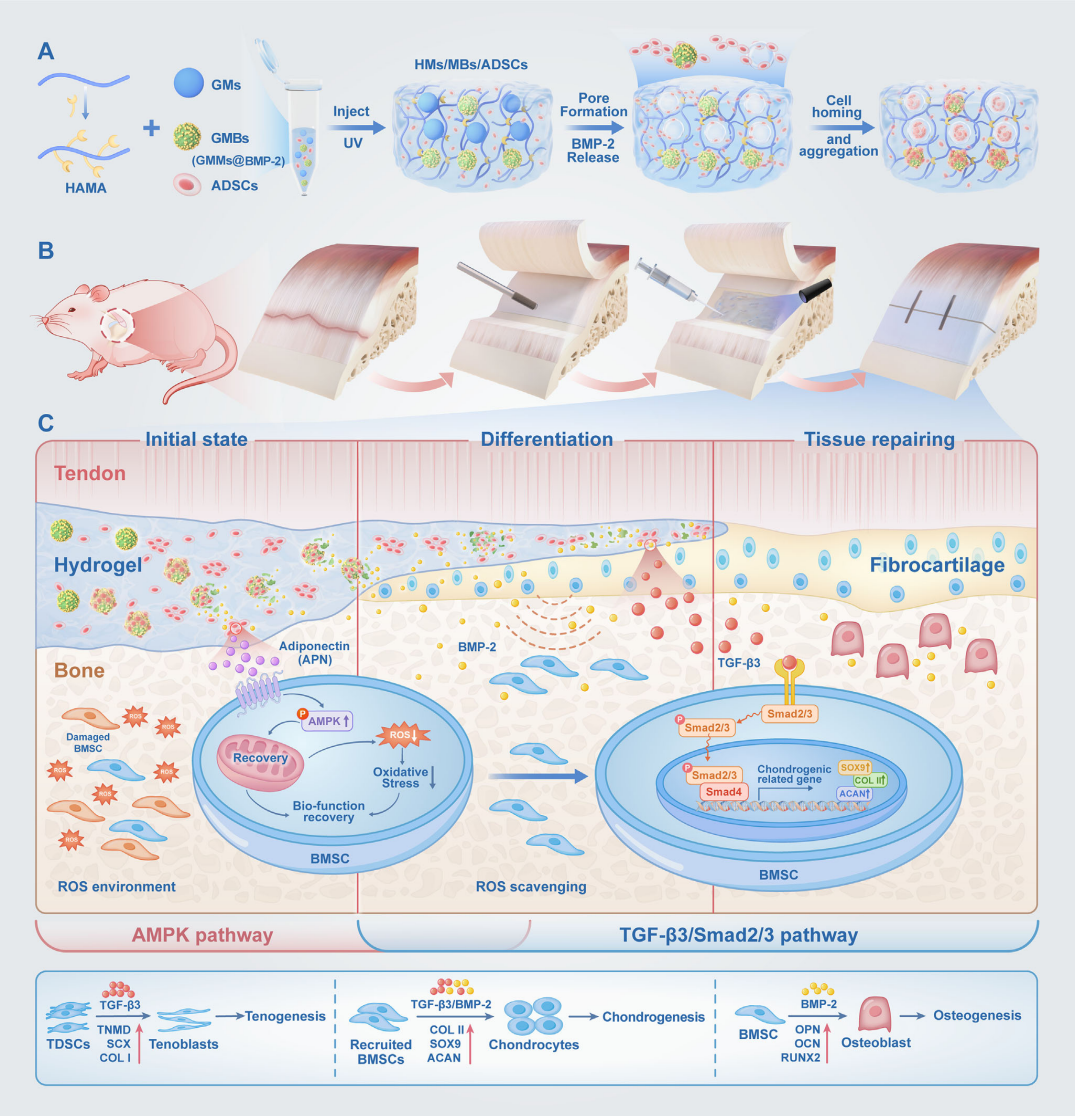
Schematic illustration of the design and application of HMs/MBs/ADSCs. A) Fabrication of the HMs/MBs/ADSCs hydrogel. After cross‐linking, micropores formed under body temperature conditions. Cells migrated to the GMBs and pore edges, eventually attaching to the GMBs and aggregating into clusters. B) Establishment of a rat rotator cuff injury model. C) Schematic representation of the mechanism mediating the reconstruction of the tendon‐to‐bone interface after HMs/MBs/ADSCs implantation.

In this study, we selected ADSCs as the seed cells. To establish a suitable microenvironment for ADSCs, we utilized the thermosensitivity of gelatin microspheres (GMs) as porogens and incorporated them into hyaluronic acid methacrylate (HAMA) to form micropores, thereby creating 3D porous hydrogel structures (HMs). Furthermore, gelatin methacryloyl (GelMA) hydrogel microspheres (GMMs) were employed to efficiently load BMP‐2. Briefly, ADSCs were mixed with HMs, and GMMs loaded with BMP‐2 (GMBs) were incorporated to develop an injectable 3D porous hydrogel cell‐loaded construct capable of sustaining BMP‐2 release (HMs/MBs/ADSCs, **Figure** **1A**). This construct was systematically characterized and applied to a rat rotator cuff injury model (Figure 1B). To explore the mechanism of this construct in the repair of the rotator cuff, in vivo and in vitro dual sequencing was performed to elucidate the signaling pathways through which BMP‐2 collaborates with ADSCs to mediate TBI repair (Figure 1C). This study aimed to facilitate TBI healing by ensuring stable BMP‐2 delivery and continuous microenvironment adaptation while providing novel insights into tissue repair pathways.

## 2 Results

### 2.1 Preparation and Characterization of Microspheres

**Figure 2:**
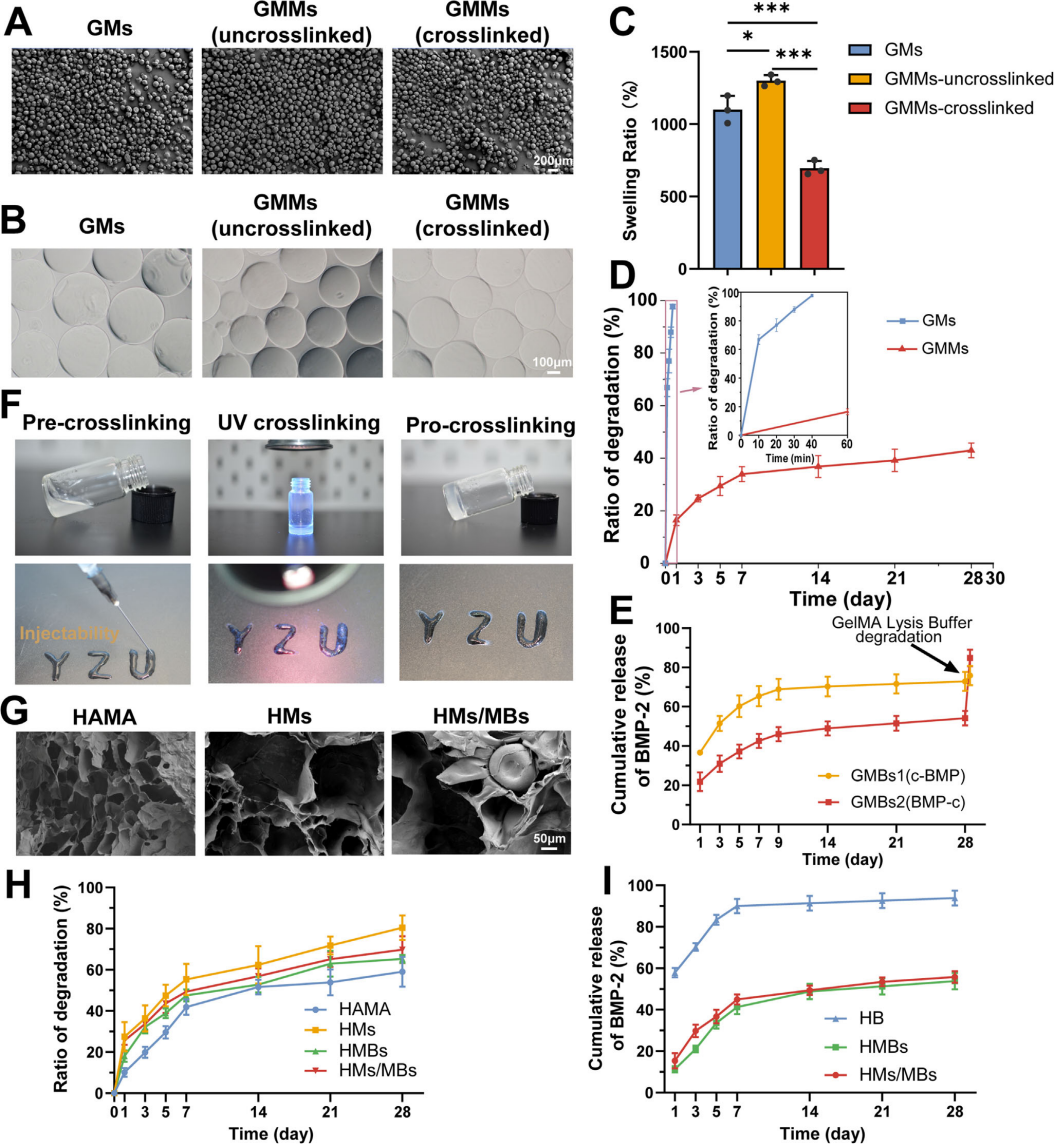
Characterization of microspheres and hydrogels. A) SEM surface images of different microspheres. B) Wet images of different microspheres under a microscope. C) 24‐hour swelling rate of microspheres. D) Post‐crosslinking degradation behavior of GMs and GMMs. E) In vitro BMP‐2 release from GMMs with different loading methods. F) Macroscopic view of HMs/MBs hydrogel before and after crosslinking. G) SEM cross‐sectional images of different hydrogels. H) Degradation behavior of hydrogels over time. I) In vitro release of BMP‐2 coated with different hydrogels. *n* = 3. *p < 0.05, **p < 0.01, ***p < 0.001.

Both types of microspheres were prepared using a double‐emulsion method. GMs were used as porogens to create pores, whereas GMMs were used to construct a sustained‐release system. The proton nuclear magnetic resonance (¹H NMR) spectra of GelMA and gelatin are shown in Figure S1 (Supporting Information). Subsequently, microspheres with diameters of 100 to 150 µm were selected using a sieve. The morphology of the screened GMs was similar to that of the GMMs, both before and after crosslinking, with regular and roughly spherical shapes (**Figure** **2**A). After swelling in water, the diameter of the microspheres was 150–200 µm (Figure 2B). Moreover, GMs (1100.48 ± 95.05%) and GMMs before crosslinking (1300.04 ± 38.85%) exhibited a higher swelling rate than that of GMMs after crosslinking (695.07 ± 50.26%) (Figure 2C), which may be because of the tighter internal network structure of GMMs post‐crosslinking. In addition, the degree of crosslinking was indirectly evaluated by calculating the swelling ratio. The swelling ratio of the crosslinked GMMs was calculated to be 7.95 ± 0.41, whereas that of the non‐crosslinked GMMs was 14 ± 0.31. The swelling ratio decreased by approximately twofold after crosslinking, indicating a significant increase in the degree of crosslinking. Owing to temperature sensitivity, GMs were almost completely degraded within 40 min, whereas the degradation rate of the crosslinked GMMs was 43 ± 2.76% after 28 days (Figure 2D). To optimize BMP‐2 release, we compared two methods of BMP‐2 incorporation into GMMs: crosslinking before loading (GMBs1) and crosslinking after loading (GMBs2), and we found that GMBs2 resulted in a slower overall release. Generally, the release was faster during the first week, with a cumulative release of 42.53 ± 3.6% for GMBs2. The release then gradually plateaued, reaching a cumulative release rate of 54.14 ± 3.68% by day 28. To confirm residual BMP‐2 in the microspheres, complete lysis was performed using GelMA lysis buffer, resulting in the release of 30.61 ± 2.2% from GMBs2, compared to only 3 ± 0.23% from GMBs1 (Figure 2E).

### 2.2 Construction of HMs/MBs Hydrogel Injection System

The ¹H NMR and Fourier‐transform infrared spectroscopy spectra of HAMA (the continuous phase of the hydrogel) and hyaluronic acid (HA) are shown in Figure S2 (Supporting Information). The characteristic peak of methacrylate (C═C bond) at 1700 cm⁻¹ confirmed its successful incorporation into HA. To determine the optimal HAMA concentration, we compared its rheological properties at concentrations of 1%, 1.5%, and 2%. The 2% concentration exhibited high viscosity while retaining the shear‐thinning behavior. Additionally, the 2% concentration exhibited a high storage modulus (G′) after UV crosslinking at a constant amplitude (*γ*) and frequency. Furthermore, it exhibited an improved elastic modulus (Figure S3, Supporting Information). Regarding cytocompatibility, no significant differences were observed in the CCK‐8 assays or cell viability among the three HAMA concentrations, and all demonstrated good biocompatibility (Figure S4, Supporting Information). Based on these findings, 2% HAMA was selected for subsequent experiments. As shown in Figure 2F, the HMs/MBs hydrogel precursor is formed by mixing GMs and GMBs into HAMA. The precursor was extruded from the syringe and formed into various shapes, transitioning from sol to gel upon UV crosslinking. By comparing the rheological differences between HAMA and GMs or GMBs, we found that they did not affect the shear‐thinning behavior of HAMA (Figure S5A, Supporting Information). The modulus after UV crosslinking exhibited a similar trend: rapid curing with a sharp increase in the storage modulus, which stabilized after complete crosslinking (Figure S5B, Supporting Information).

### 2.3 Characterization of HMs/MBs Porous Hydrogel

**Figure 3:**
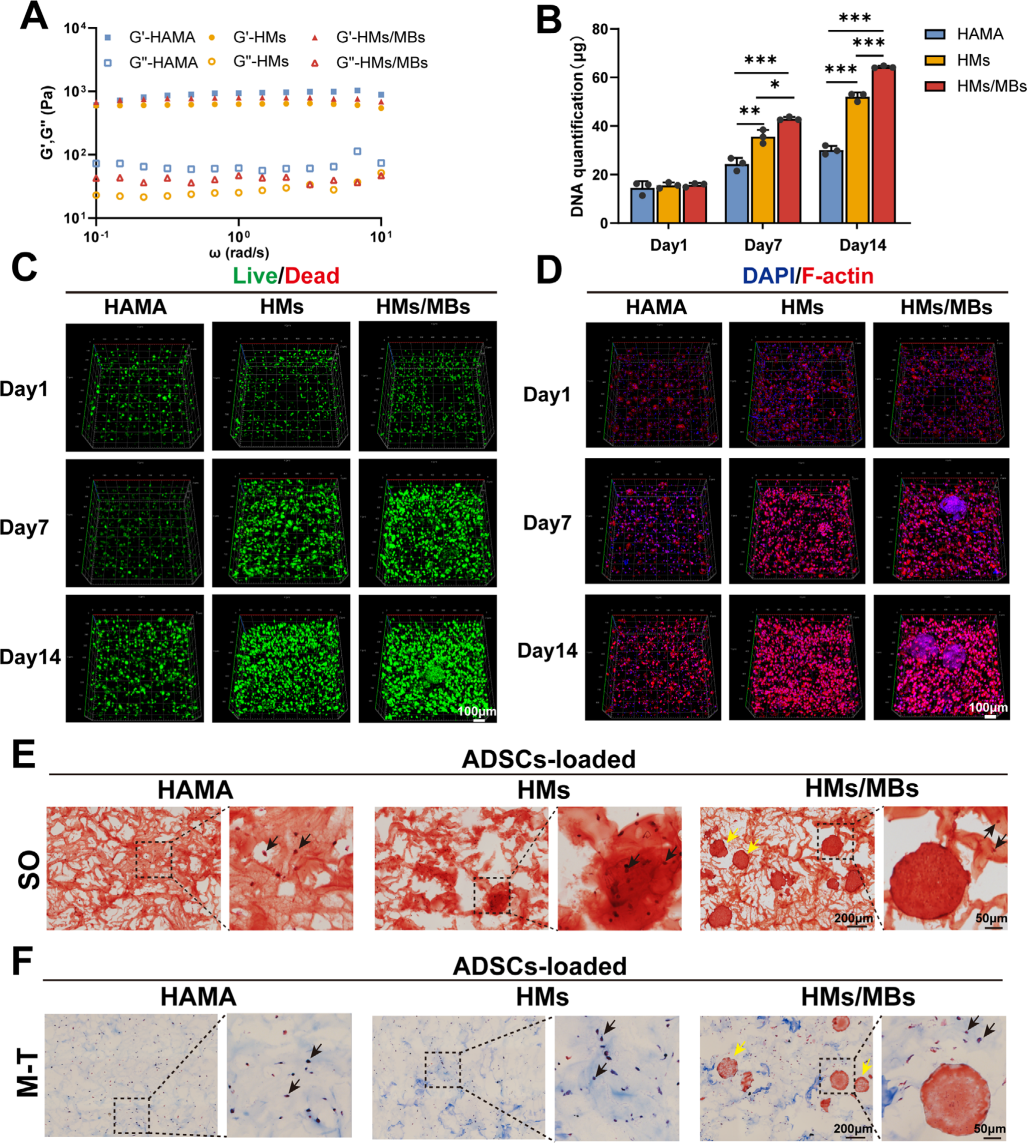
Dynamic modulus of porous hydrogels and effects of the 3D environment of different hydrogels on ADSCs. A) Dynamic modulus from 10^−1^ to 10 *ω* at a constant oscillation amplitude (*γ*) of 1%. B) DNA quantification of ADSCs encapsulated within different hydrogels. C) Confocal images of live/dead staining of ADSCs coated in different hydrogels. D) Confocal images of nuclear and cytoskeletal staining of ADSCs coated in different hydrogels. E) Safranin O staining of ADSCs encapsulated in different hydrogels. F) Masson's trichrome staining of ADSCs encapsulated in different hydrogels. GMBs indicated by yellow arrows; ADSCs indicated by black arrows. *n* = 3.*p < 0.05, **p < 0.01, ***p < 0.001.

Upon UV exposure, the injected hydrogel crosslinked, with HAMA exhibiting a uniform pore size of 50 to 100 µm. The addition of GMs increased the leaching pore size to ≈200 µm. Embedded GMBs were observed via scanning electron microscopy (SEM) (Figure 2G) of HMs/MBs without affecting the leaching pores. As shown in Figure 2H, the porous hydrogels (HMs) degraded at a faster rate, with an initial rapid phase followed by a slower phase; the degradation of HMs/MBs reached 69.97 ± 6.56% by day 28. As shown in Figure 2I, direct loading of BMP‐2 into HAMA (HB) resulted in a burst release, whereas controlled release was achieved through GMMs encapsulation (HMBs), with a cumulative release of 53.76 ± 3.92% by day 28. The pores formed by GMs had minimal impact on BMP‐2 release, with a cumulative release of 55.82 ± 2.76% for HMs/MBs. From a rheological perspective, the addition of GMs and GMMs had a minimal effect on the storage modulus at constant frequency and strain (**Figure** **3**A; Figure S5C, Supporting Information). However, the stress–strain curve showed that under a strain of 45%, the three groups maintained a certain toughness (Figure S5D, Supporting Information). At 15% strain, the compression modulus was 24.7 ± 1.84 kPa for HAMA, 13.14 ± 1.96 kPa for HMs, and 15.23 ± 2.47 kPa for HMs/MBs (Figure S5E, Supporting Information). The pores leached from the GMs increased both the rate and extent of hydrogel swelling. HAMA (1647.45 ± 153.33%) reached swelling equilibrium in 30 min, whereas HMs (2315.08 ± 122.68%) and HMs/MBs (2125.05 ± 124.96%) reached equilibrium in 90 min (Figure S6, Supporting Information).

To investigate the 3D microenvironment created by HMs/MBs, ADSCs were uniformly mixed into the precursor before gelation, thereby being encapsulated within the hydrogel. DNA was extracted from the ADSCs‐encapsulated hydrogel after dissociation on days 1, 7, and 14. As shown in Figure 3B, there were no significant differences among the three groups on day 1; however, the HMs/MBs group showed significantly high levels on days 7 and 14. The confocal microscopy results of live/dead staining revealed that the proliferation rate of ADSCs in the HMs and HMs/MBs groups was higher than that in the HAMA group. Additionally, cell aggregation was observed on day 7, and significant aggregation was observed on day 14. By day 14, the aggregation in the HMs/MBs group was the most pronounced, forming cell clusters. This suggested that the addition of GMs provided space for cell proliferation, whereas BMP‐2 further promoted cell proliferation and aggregation (Figure 3C). Confocal images of nuclear and cytoskeletal staining yielded results similar to those of the live/dead staining. The difference lies in the fact that DAPI and actin stain GMBs, thereby labeling the GMBs (Figure 3D; Figure S7, Supporting Information). It was more clearly observed that ADSCs aggregated around the GMBs, indicating that BMP‐2 might have played a role in attracting cell migration. Cryosections of the ADSCs‐loaded hydrogel constructs incubated for 14 days were prepared and stained with safranin O and Masson's trichrome. Compared with the cell‐free hydrogels (Figure S8, Supporting Information), ADSCs within the hydrogels (indicated by black arrows) and GMBs in the HMs/MBs group (indicated by yellow arrows) were clearly visible (Figure 3E,F). Owing to the addition of the pore‐forming GMs, the pore size of the hydrogels in the HMs and HMs/MBs groups was slightly larger than that in the HAMA group, as seen in the safranin O images. However, this difference was not highly pronounced, likely because some extracellular matrices secreted by the cells filled the internal network of the hydrogels by day 14.

### 2.4 In Vivo Biosafety Assessment of HMs/MBs/ADSCs Hydrogels: Cell Tracking, Degradation Behavior, and Short‐Term Inflammatory Response

At 2 weeks post‐surgery, H&E staining of the heart, liver, spleen, lungs, and kidneys of the rats indicated no adverse effects on major organs in either the experimental or control groups (Figure S10, Supporting Information). Additionally, the blood routine and liver/kidney function tests of the HMs/MBs and HMs/MBs/ADSCs hydrogel groups showed no statistically significant differences compared with those of the blank control group (Figure S11, Supporting Information). Therefore, the in vivo implantation of HMs/MBs/ADSCs hydrogels is biologically safe. From the in vivo subcutaneous degradation experiments, it was observed that neither hydrogel group fully degraded after 4 weeks. However, the HMs/MBs/ADSCs hydrogel degraded faster than the HMs/MBs hydrogel (Figure S12, Supporting Information). The retention of ADSCs is crucial to their paracrine effects. Small‐animal in vivo imaging results demonstrated that ADSCs encapsulated in the HMs/MBs hydrogels were retained for at least 6 weeks post‐surgery, although the fluorescence intensity decreased over time (Figure S13, Supporting Information). At 2 weeks post‐surgery, CD68 and CD163 immunofluorescence staining was performed on tissues from the three groups to identify M1 and M2 macrophages (M*φ*), respectively (Figure S14, Supporting Information). The study found that hydrogel groups with better tissue regeneration and immunomodulatory capabilities have the potential to polarize M*φ* into the pro‐regenerative M2 phenotype.^[^ ^24^ ^]^ The control group exhibited the highest number of M1 M*φ* and the lowest number of M2 M*φ*, indicating an acute inflammatory response in the surgical area. By contrast, the hydrogel groups demonstrated varying degrees of immunomodulatory ability, with reduced levels of acute inflammation compared with those of the control group. The HMs/MBs/ADSCs group showed more M2 M*φ* and fewer M1 M*φ* than those of the HMs/MBs group.

### 2.5 Biomechanical and Bone Repair Effects of HMs/MBs/ADSCs in a Rat Rotator Cuff Tear Model

**Figure 4:**
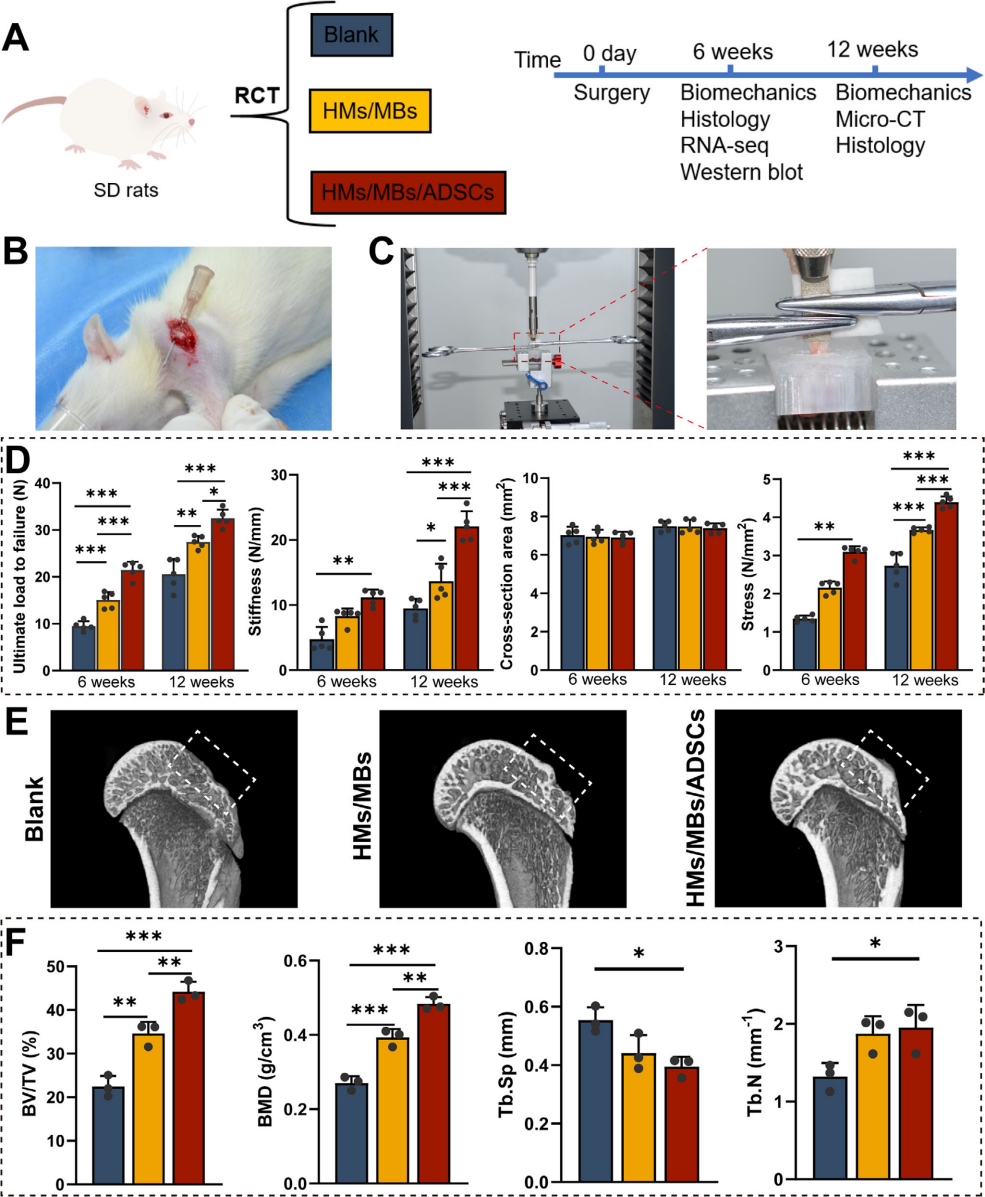
Biomechanical properties and micro‐CT analysis. A) Diagram illustrating of the animal experimental design. B) Location of supraspinatus tendon. C) Stretching diagram of supraspinatus tendon‐humeral tissue. D) Tensile test analysis of ultimate load to failure, stiffness, cross‐sectional area, and stress. *n* = 5. E) Micro‐CT scans of the proximal humerus. F) Quantitative micro‐CT analysis of BV/TV, BMD, Tb.Sp, and Tb.N. *n* = 3. *p < 0.05, **p < 0.01, ***p < 0.001.

Rats were assigned to three groups as illustrated in **Figure** **4**A. Biomechanical tests were performed on the regenerated supraspinatus tendon–humerus complexes at 6 and 12 weeks (Figure 4C). No significant differences were observed in the cross‐sectional areas of the supraspinatus tendon (Figure 4D). At 6 weeks, the maximum failure load was significantly higher in the HMs/MBs/ADSCs (21.46 ± 1.76 N) and HMs/MBs (15.05 ± 1.73 N) groups compared with that in the blank group (9.48 ± 1.04 N). Stiffness (11.16 ± 1.2 N mm^−1^) and stress (3.1 ± 0.14 mm⁻¹) in the HMs/MBs/ADSCs group were also higher than in the blank group (stiffness: HMs/MBs, 8.28 ± 1.18 N mm^−1^; blank, 4.74 ± 1.89 N mm^−1^; stress: HMs/MBs, 2.16 ± 0.16 mm⁻¹, blank, 1.35 ± 0.08 mm⁻¹). At 12 weeks, the trend in maximum failure load was similar to that at 6 weeks, with stiffness and stress significantly higher in both experimental groups than in the blank group (Ultimate load to failure: HMs/MBs/ADSCs, 32.48 ± 1.89 N; HMs/MBs, 27.44 ± 1.31 N; blank, 20.54 ± 3.3 N; stiffness: HMs/MBs/ADSCs, 22.08 ± 2.33 N mm^−1^; HMs/MBs, 13.63 ± 2.71 N mm^−1^; blank, 9.49 ± 1.45 N mm^−1^; stress: HMs/MBs/ADSCs, 4.4 ± 0.15 mm⁻¹; HMs/MBs, 3.67 ± 0.07 mm⁻¹; blank, 2.73 ± 0.35 mm⁻¹). The HMs/MBs/ADSCs group exhibited the best overall biomechanical properties, more than twice that of the blank group. At 12 weeks post‐surgery, new bone formation in the humeral head was further evaluated using micro‐CT (Figure 4E); 3D reconstruction indicated that new bone regeneration in the HMs/MBs/ADSCs group was more substantial and complete. Bone volume/total volume (BV/TV), bone mineral density (BMD), trabecular number (Tb.N), and trabecular separation (Tb.Sp) were semi‐quantitatively analyzed (Figure 4F). At 12 weeks, BV/TV and BMD in the experimental groups were significantly higher than in the blank group (BV/TV [%]: HMs/MBs/ADSCs, 44.17 ± 2.3; HMs/MBs, 34.61 ± 2.65; blank, 22.44 ± 2.45; BMD (g cm^−^^3^): HMs/MBs/ADSCs, 0.48 ± 0.02; HMs/MBs, 0.39 ± 0.02; blank, 0.27 ± 0.02). Additionally, compared with observations in the blank group, Tb.Sp in HMs/MBs/ADSCs was lower, whereas Tb.N was higher (Tb.Sp (mm): HMs/MBs/ADSCs, 0.4 ± 0.03; HMs/MBs, 0.44 ± 0.06; blank, 0.55 ± 0.04; Tb.N (mm⁻¹): HMs/MBs/ADSCs, 1.95 ± 0.29, HMs/MBs, 1.87 ± 0.23, blank, 1.32 ± 0.17).

### 2.6 Fibrocartilage and Tendon Regeneration Induced by HMs/MBs/ADSCs In Vivo

**Figure 5:**
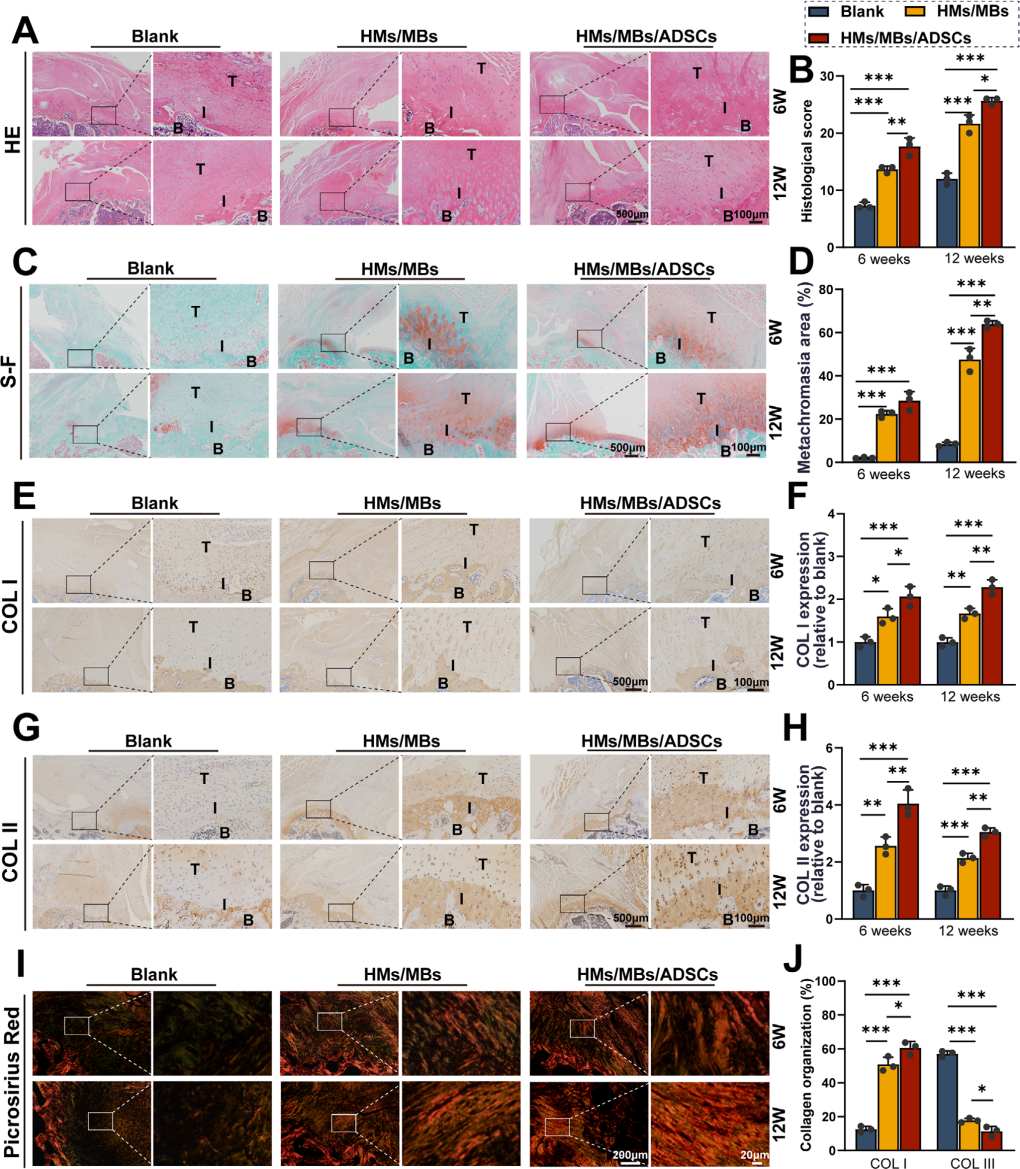
Morphological analysis of newly formed TBI at 6 and 12 weeks after rotator cuff repair. A) H&E staining of TBI. B) Histological score of TBI. C) Safranin fast green staining of TBI. D) Semi‐quantitative analysis of the metachromatic areas of safranin fast green staining. E–H) Immunohistochemical staining images and semi‐quantitative analysis of COL I and COL II. I) Picrosirius red staining of TBI. J) Semi‐quantitative analysis of collagen composition after 12 weeks in Picrosirius red staining. T: tendon; I: interface; B: bone. *n* = 3. *p < 0.05, **p < 0.01, ***p < 0.001.

According to H&E staining (**Figure** **5**A) and Masson's trichrome staining (Figure S15, Supporting Information), the blank group exhibited higher cell density and a more disorganized cell arrangement than those of the two experimental groups at 6 weeks. Tendon collagen fibers in the blank group appeared disorganized and did not align toward the bone. By contrast, the collagen fibers in the experimental groups showed an initial alignment, with the HMs/MBs/ADSCs group demonstrating a slightly more organized arrangement than that of the HMs/MBs group. At 12 weeks, the cell density decreased across all groups; however, collagen fibers in the blank group remained disorganized, hindering tendon regeneration. Collagen fibers were organized in the experimental groups; however, the distal end of the HMs/MBs supraspinatus tendon was slightly disorganized, whereas the HMs/MBs/ADSCs group exhibited a highly organized arrangement, leading to superior tendon regeneration. Therefore, the histological scores of the HMs/MBs/ADSCs group were significantly higher than those of the other groups (Figure 5B). Fibrocartilage regeneration was evaluated by safranin fast green staining (Figure 5C,D) and immunohistochemical staining for COL I and COL II (Figure 5E–H). At 6 and 12 weeks, tendon–bone fusion in the blank group remained poor, with a distinct separation at the boundary. Fibrocartilage‐like tissue formation was evident in both experimental groups at 6 weeks, with interface fusion beginning. Chondrocytes in the HMs/MBs/ADSCs group were arranged more neatly than those in the HMs/MBs group. At 12 weeks, chondrocytes in the HMs/MBs/ADSCs group exhibited a more regular arrangement and achieved the best interface fusion. Consequently, COL I and COL II expression levels were significantly high in the HMs/MBs/ADSCs group. Picrosirius red staining further confirmed the formation of more mature regenerated tendons in the HMs/MBs/ADSCs group at both 6 and 12 weeks, characterized by high collagen type I and low collagen type III contents (Figure 5I,J; Figure S16, Supporting Information).

### 2.7 Mechanism by Which HMs/MBs and HMs/MBs/ADSCs Promote TBI Regeneration Explored by In Vivo Transcriptome Sequencing

**Figure 6:**
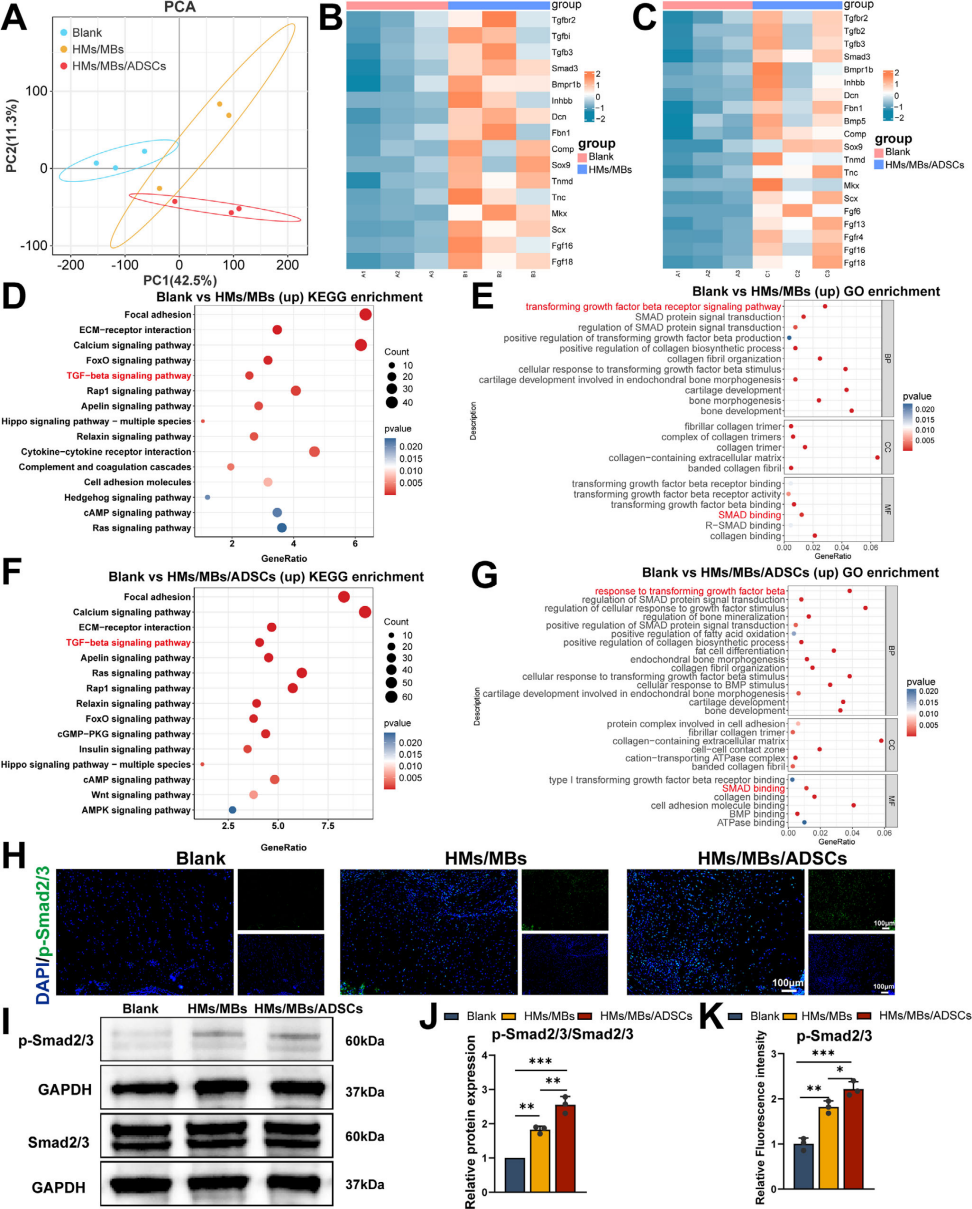
Transcriptome sequencing and related pathway analysis of tissue healing regulated in vivo by composite hydrogels with or without cells. A) PCA of all samples in the Blank, HMs/MBs, and HMs/MBs/ADSCs groups. B,C) Heatmaps of genes associated with tissue repair. D,F) KEGG enrichment analysis of DEGs. E,G) GO enrichment analysis of DEGs. H,K) Immunofluorescence staining and semi‐quantitative analysis for p‐Smad2/3. I,J) Western blot and semi‐quantitative analysis of Smad2/3 and p‐Smad2/3. *n* = 3. *p < 0.05, **p < 0.01, ***p < 0.001.

To elucidate the molecular mechanisms by which HMs/MBs/ADSCs regulate TBI healing, we employed RNA sequencing to systematically analyze relevant gene expression profiles. Principal component analysis (PCA) was first conducted to assess homogeneity and heterogeneity among the experimental groups (**Figure** **6**A). The results showed that PC1 and PC2 accounted for 42.5% and 11.3% of the total variance, respectively. The blank and treatment groups (HMs/MBs and HMs/MBs/ADSCs) exhibited significant separation along the PC1 axis, indicating that the treatment conditions had a substantial impact on gene expression profiles. Additionally, the distribution differences between the HMs/MBs and HMs/MBs/ADSCs groups along the PC1 and PC2 axes suggest that the incorporation of ADSCs may exert additional regulatory effects on gene expression. No significant outliers were observed, confirming the reliability of the data for subsequent analyses. Based on differential expression analysis, volcano plot results (Figure S17A,B, Supporting Information) revealed that, compared with the blank group, the HMs/MBs group had 4780 significantly upregulated and 3048 significantly downregulated genes, whereas the HMs/MBs/ADSCs group exhibited 7512 upregulated and 6920 downregulated genes. Further heatmap analysis of genes related to TBI healing (Figure 6B,C) demonstrated that, compared with the blank group, both treatment groups exhibited a significant upregulation trend in genes associated with osteochondral and tendon healing. To explore potential signaling pathways, the Kyoto Encyclopedia of Genes and Genomes (KEGG) pathway enrichment analysis was performed (Figure 6D,F). The results indicated that the transforming growth factor‐beta (TGF‐β) signaling pathway was significantly upregulated in both the HMs/MBs and HMs/MBs/ADSCs groups compared with that in the blank group. Combined with gene heatmap analysis, a notable increase in Smad3 expression was observed, suggesting that the TGF‐β/Smad2/3 signaling pathway may play a critical role in this process. This finding was consistent with Gene Ontology functional enrichment analysis, further supporting the importance of the TGF‐β/Smad2/3 pathway in TBI healing (Figure 6E,G). To validate these findings, immunofluorescence staining and western blotting were used to detect key molecules of the TGF‐β/Smad2/3 signaling pathway in tissue samples. Immunofluorescence results (Figure 6H,K) showed that, compared with that in the blank group, p‐Smad2/3 expression was significantly enhanced in both the HMs/MBs and HMs/MBs/ADSCs groups, with the HMs/MBs/ADSCs group exhibiting a more pronounced effect. Western blot results were consistent with the immunofluorescence findings (Figure 6I,J), demonstrating a marked upregulation of p‐Smad2/3 protein levels in the HMs/MBs (1.83, *p *< 0.01) and HMs/MBs/ADSCs (2.55, *p *< 0.001) groups compared with those in the blank group.

### 2.8 Possible Regulatory Mechanism of BMP‐2 and ADSCs‐Loaded Porous Hydrogel Explored In Vitro

**Figure 7:**
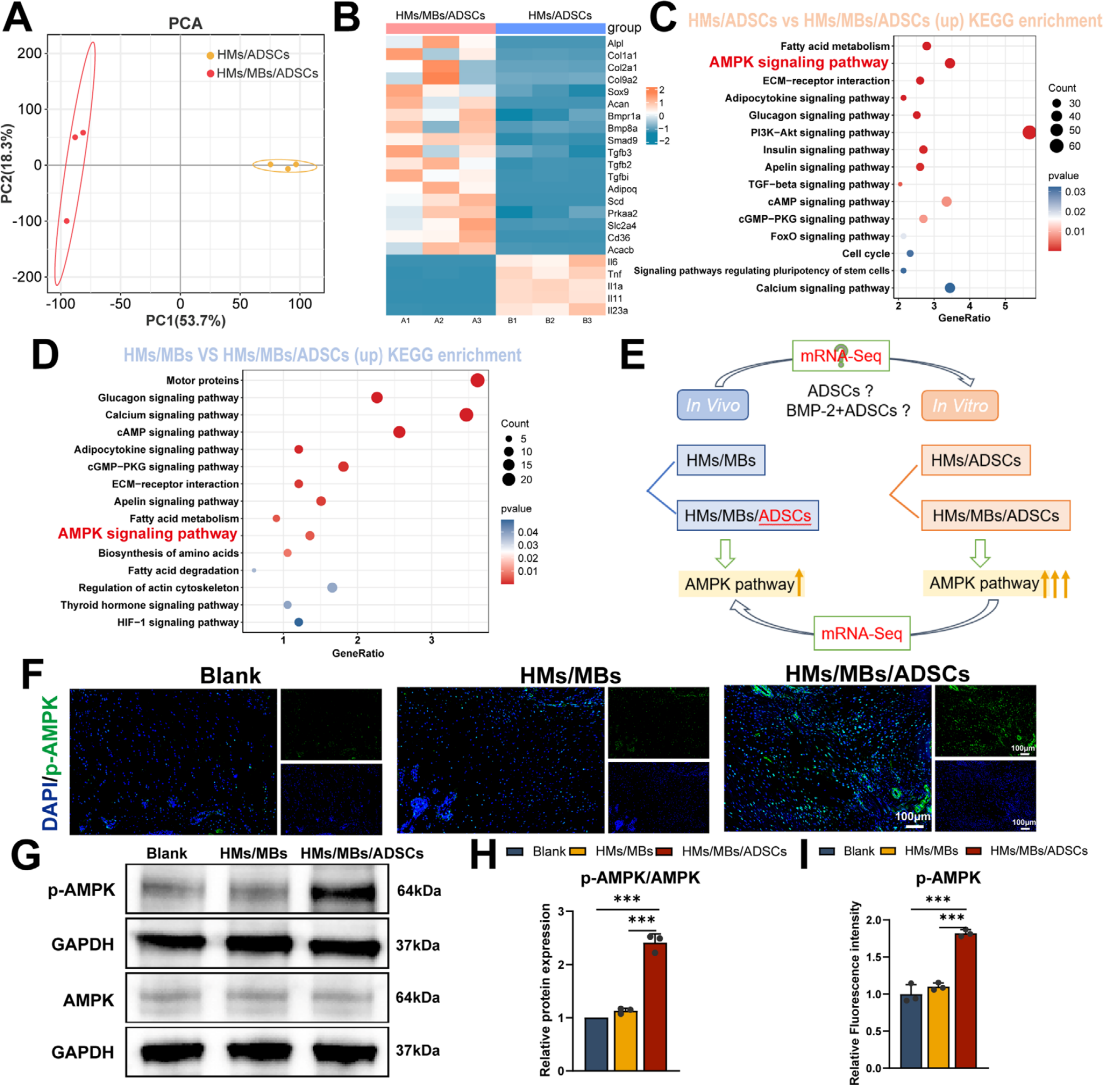
Secondary analysis of in vivo transcriptomes, pathway validation, and in vitro transcriptome sequencing of HMs/ADSCs and HMs/MBs/ADSCs. A) PCA of all samples from HMs/ADSCs and HMs/MBs/ADSCs groups. B) Heatmap of genes associated with cell differentiation and inflammation. C) KEGG enrichment analysis of DEGs in vitro. D) KEGG enrichment analysis of DEGs in vivo. E) Diagram of selecting the AMPK signaling pathway as a critical pathway. F,I) Immunofluorescence staining and semi‐quantitative analysis for p‐AMPK. G,H) Western blot and semi‐quantitative analysis of AMPK and p‐AMPK. *n* = 3. *p < 0.05, **p < 0.01, ***p < 0.001.

During in vivo sequencing analysis, a critical question emerged: whether ADSCs play an independent role in this process or function synergistically with BMP‐2 in distinct ways. To investigate this further, based on Figure 3, ADSCs were loaded into HMs and HMs/MBs hydrogel precursors. After cross‐linking, HMs/ADSCs and HMs/MBs/ADSCs hydrogels were obtained. An in‐depth in vitro RNA sequencing study was subsequently conducted. PCA revealed that PC1 and PC2 accounted for 53.7% and 18.3% of the total variance, respectively (**Figure** **7**A). Notably, the HMs/ADSCs and HMs/MBs/ADSCs groups exhibited significant separation along the PC1 axis, indicating that MBs had a substantial impact on gene expression, whereas variation along the PC2 axis may reflect minor differences within or between groups. No significant outliers were observed, confirming the high data quality. Volcano plot analysis demonstrated that, compared with the HMs/MBs group, the HMs/MBs/ADSCs group had 6906 significantly upregulated and 5370 significantly downregulated genes (Figure S18A, Supporting Information). Further heatmap analysis revealed that compared with observations in the HMs/ADSCs group, genes related to osteochondral and tendon healing were significantly upregulated in the HMs/MBs/ADSCs group, whereas inflammation‐related genes were significantly downregulated (Figure 7B). To explore potential signaling pathways, KEGG pathway enrichment analysis was performed, revealing that the AMPK pathway was significantly upregulated in the HMs/MBs/ADSCs group (Figure 7C). Notably, consistent with the in vitro results, the AMPK pathway also exhibited an upregulation trend in tissue samples from the HMs/MBs/ADSCs group compared with those from the HMs/MBs group (Figure 7D). These results suggest that the AMPK pathway plays a critical role in the synergistic effects of BMP‐2 and ADSCs (Figure 7E). To validate the in vivo sequencing findings, immunofluorescence staining, and western blotting were used to detect key molecules of the AMPK signaling pathway in tissue samples. Immunofluorescence results showed that p‐AMPK expression was significantly enhanced in the HMs/MBs/ADSCs group compared with that in the blank and HMs/MBs groups (Figure 7F,I). Western blotting results further confirmed this observation, demonstrating a marked upregulation of p‐AMPK protein levels in the HMs/MBs/ADSCs group (2.41, *p* < 0.001) compared with those in the blank and HMs/MBs groups (Figure 7G,H).

### 2.9 Validation of Mechanisms by Which HMs/MBs/ADSCs Regulate Bone Marrow‐Derived Stem Cells (BMSCs) Function through Paracrine Effects

**Figure 8:**
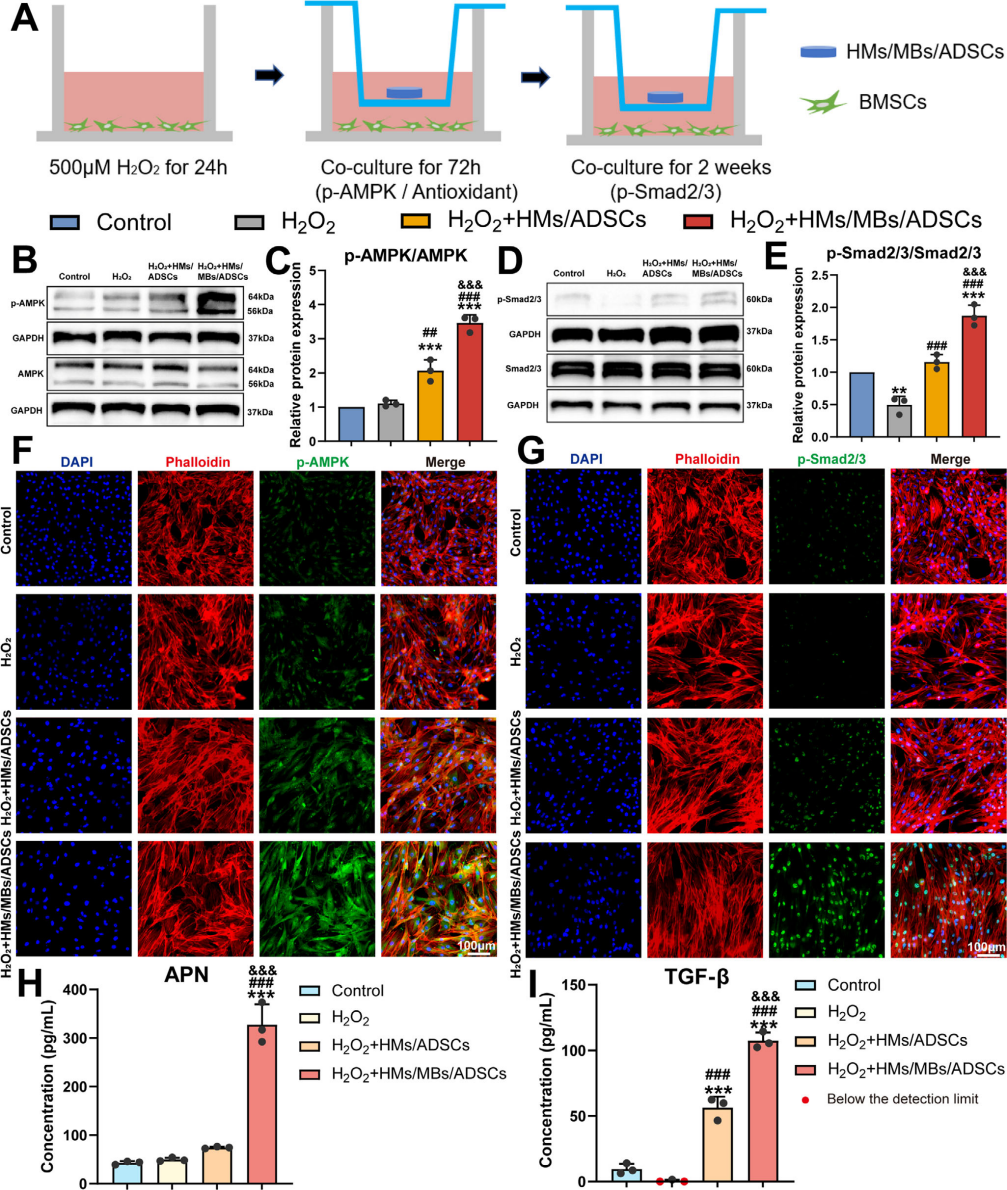
Validation of the mechanism of how HMs/MBs/ADSCs regulate BMSCs function through paracrine effects. A) Schematic illustration of experimental design. B,C) Western blot and semi‐quantitative analysis of AMPK and p‐AMPK. D,E) Western blot and semi‐quantitative analysis of Smad2/3 and p‐Smad2/3. F,G) Immunofluorescence staining of p‐AMPK and p‐Smad2/3. H) The concentration of APN was measured by ELISA. I) The concentration of TGF‐β3 was measured by ELISA. *n* = 3. *p < 0.05, **p < 0.01, ***p < 0.001, when compared with control group. ^#^ *p* < 0.05, ^##^ *p* < 0.01, ^###^ *p* < 0.001, when compared with H_2_O_2_ group. ^&^ *p* < 0.05, ^&&^ *p* < 0.01, ^&&&^ *p* < 0.001, when compared with H_2_O_2_+HMs/ADSCs group.

AMPK can mitigate mitochondrial damage caused by ROS and reduce ROS levels, thereby exerting antioxidant effects.^[^ ^25^ ^]^ Therefore, it is hypothesized that ADSCs activate the AMPK pathway via paracrine signaling, thereby preventing oxidation. To simulate the in vivo microenvironment and verify the expression of the relevant pathways, an in vitro oxidative stress model was constructed. BMSCs, the most enriched cells at the interface, were selected as host cells (**Figure** **8**A). To further validate this experimental condition, CCK‐8 assay results showed that after 24 h of H₂O₂ stimulation, BMSCs viability showed no statistically significant difference compared with that in the control group. Additionally, after switching to a complete culture medium, the cells were still able to proliferate rapidly, although differences in proliferation were observed compared with the proliferation in the control group (Figure S19A, Supporting Information). The live/dead assay results indicated that the morphology of the stimulated cells was slightly different from that of the control group, with almost no significant number of dead cells (Figure S19B, Supporting Information). The AMPK pathway was verified using western blotting (Figure 8B,C) and immunofluorescence (Figure 8F; Figure S20A, Supporting Information). Both showed consistent results, with significantly higher p‐AMPK protein expression in the H₂O₂+HMs/MBs/ADSCs and H₂O₂+HMs/ADSCs groups than in the control and H₂O₂ groups. This effect was more pronounced in the H₂O₂+HMs/MBs/ADSCs group (H₂O₂+HMs/MBs/ADSCs versus H₂O₂+HMs/ADSCs in WB: 3.46 vs 2.07, *p* < 0.001). Additionally, the in vitro expression of the TGF‐β signaling pathway was further validated in vivo. Western blotting (Figure 8D,E) and immunofluorescence (Figure 8G; Figure S20B, Supporting Information) demonstrated that HMs/MBs/ADSCs significantly increased p‐Smad2/3 protein expression (H₂O₂+HMs/MBs/ADSCs versus Control in WB: 1.87, *p* < 0.001), whereas the H₂O₂ group showed a significant decrease (H₂O₂ vs Control in WB: 0.57, *p* < 0.01). Subsequently, the specific paracrine factors that activate both pathways were investigated. Transcriptome data were further analyzed by differentially expressed genes, combined with a literature review, identifying two factors: adiponectin (APN) and TGF‐β3 (Figure 7B; Figure S18A, Supporting Information). ELISA results indicated significantly higher APN concentrations in the H₂O₂+HMs/MBs/ADSCs group (327.78 ± 41.55 pg mL^−1^) compared with the other groups (APN (pg mL^−1^): H₂O₂+HMs/ADSCs: 74.49 ± 2, H₂O₂: 49.67 ± 3.84, Control: 43.11 ± 3.58) (Figure 8H). TGF‐β3 concentrations significantly increased in both the H₂O₂+HMs/MBs/ADSCs (107.47 ± 6.25 pg mL^−1^) and H₂O₂+HMs/ADSCs (56.25 ± 8.48 pg mL^−1^) groups, compared with those in the H₂O₂ (below the detection limit) and Control (9.62 ± 3.97 pg mL^−1^) groups (Figure 8I).

### 2.10 Evaluation of Antioxidant Effects of HMs/MBs/ADSCs In Vitro

**Figure 9:**
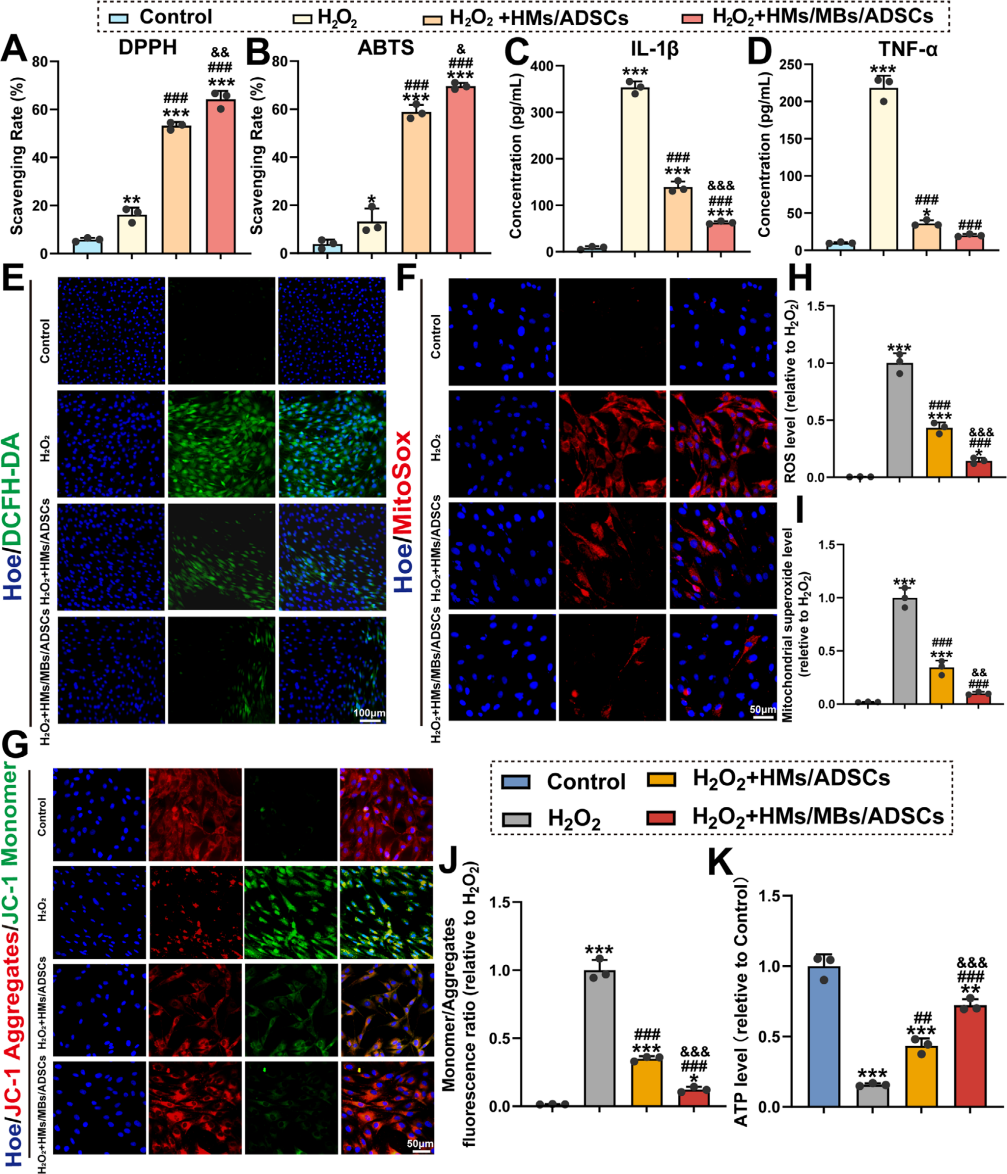
HMs/MBs/ADSCs alleviated the H_2_O_2_‐induced oxidative stress of BMSCs. A,B) Scavenging rate of DPPH and ABTS free radical. C,D) The concentration of IL‐1β and TNF‐α was measured by ELISA. E) Representative DCFH‐DA staining images for intracellular ROS. F) Representative MitoSOX staining images. G) Immunofluorescence images of mitochondrial membrane potential (*ΔΨm*) detected by JC‐1 probe. H–J) Semiquantitative analysis of the relative fluorescent intensity of (E)–(G). K) ATP levels in BMSCs measured by an ATP Assay Kit. *n* = 3. *p < 0.05, **p < 0.01, ***p < 0.001, when compared with control group. ^#^ *p* < 0.05, ^##^ *p* < 0.01, ^###^ *p* < 0.001, when compared with H_2_O_2_ group. ^&^ *p* < 0.05, ^&&^ *p* < 0.01, ^&&&^ *p* < 0.001, when compared with H_2_O_2_+HMs/ADSCs group.

Mitochondria generate ROS as byproducts of the respiratory chain. While moderate levels maintain cellular functions, excessive ROS damage mitochondria.^[^ ^26^ ^]^ To verify the antioxidant effect of HMs/MBs/ADSCs, this study constructed an oxidative stress model based on ROS upregulation during the inflammatory phase, which induces increased cellular ROS production (Figure 8A). Free radical scavenging in the supernatant revealed that the HMs/MBs/ADSCs (64.22 ± 3.5%) and HMs/ADSCs (53.2 ± 1.55%) groups exhibited higher DPPH radical scavenging rates compared with those of the H₂O₂ group (16.15 ± 2.94%) (**Figure** **9**A). Similarly, the HMs/MBs/ADSCs (69.62 ± 1.34%) and HMs/ADSCs (58.86 ± 3.01%) groups showed higher ABTS radical scavenging rates than those of the H₂O₂ group (13.24 ± 5.39%) (Figure 9B). Free radical scavenging assays demonstrated that the hydrogels effectively cleared DPPH and ABTS radicals, achieving antioxidant effects. Additionally, the concentration of proinflammatory factors (interleukin‐1β [IL‐1β] and tumor necrosis factor [TNF]‐α) in the supernatant was measured (Figure 9C,D). The results indicated that the HMs/MBs/ADSCs and HMs/ADSCs groups had lower levels of proinflammatory factors compared with those of the H₂O₂ group, with the HMs/MBs/ADSCs group showing more pronounced effects (IL‐1β: 62.94 ± 2.92 pg mL^−1^ for HMs/MBs/ADSCs, 139.24 ±  12.04 pg mL^−1^ for HMs/ADSCs, 353.55 ± 13.06 pg mL^−1^ for H₂O₂; TNF‐α: 20.09 ± 1.79 pg mL^−1^ for HMs/MBs/ADSCs, 36.24 ± 4.08 pg mL^−1^ for HMs/ADSCs, 218.53 ± 16.07 pg mL^−1^ for H₂O₂). Total ROS and superoxide levels were quantified using DCFH‐DA and MitoSOX reagents, respectively. Compared with that in the H₂O₂ group, DCFH‐DA fluorescence intensity was markedly reduced in the H₂O₂+HMs/MBs/ADSCs (0.14, *p* < 0.001) and H₂O₂+HMs/ADSCs (0.43, *p* < 0.001) groups (Figure 9E,H). Moreover, superoxide levels in the H₂O₂+HMs/MBs/ADSCs (0.10, *p* < 0.001) and H₂O₂+HMs/ADSCs (0.34, *p* < 0.001) groups were significantly reduced (Figure 9F,I). Therefore, HMs/ADSCs and HMs/MBs/ADSCs hydrogels effectively cleared excess ROS. Excess ROS accumulation disrupts mitochondrial structure and alters membrane potential. The H₂O₂ group exhibited significantly high levels of JC‐1 monomers (Figure 9G). Both HMs/MBs/ADSCs (0.12, *p* < 0.001) and HMs/ADSCs (0.35, *p* < 0.001) effectively stabilized *ΔΨm*, with the effect being more pronounced in the HMs/MBs/ADSCs group (*p* < 0.001) (Figure 9J). The H₂O₂ group showed a significant decrease in membrane potential, which stabilized after hydrogel intervention. Mitochondria are the primary sites for ATP production. To indirectly assess mitochondrial function, ATP levels were further measured. The results indicated that HMs/MBs/ADSCs effectively restored ATP production (H₂O₂+HMs/MBs/ADSCs vs H₂O₂: 0.72 vs 0.16, *p* < 0.001) (Figure 9K). These results suggest that BMP‐2 and ADSCs act synergistically. The AMPK pathway is activated at an early stage, leading to a reduction in ROS production. An antioxidant effect is achieved in BMSCs, thereby restoring their biological functions.

### 2.11 HMs/MBs/ADSCs Enhanced Proliferation and Migration of BMSCs and Promoted Differentiation of Both BMSCs and Tendon‐Derived Stem Cells (TDSCs)

**Figure 10:**
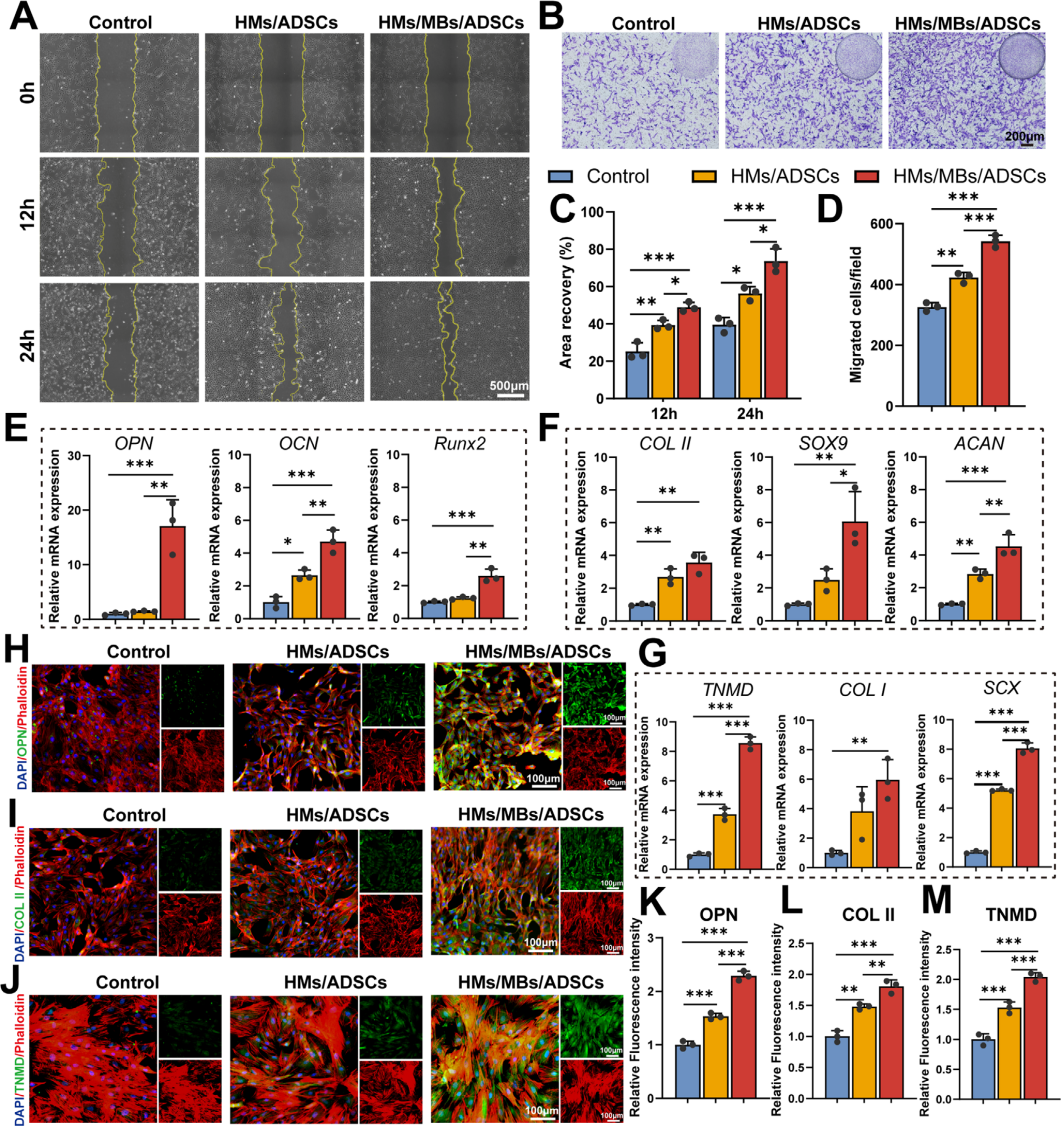
HMs/MBs/ADSCs enhance the migration of BMSCs and facilitate the differentiation of both BMSCs and TDSCs. A) Microscopy images of the scratch wounds at 0, 12, and 24 h. B) Transwell assay of BMSCs migration. C) Quantitative analysis of the recovery area in the scratch assay. D) Quantitative analysis of BMSC migration counts in the Transwell assay. E) RT‐qPCR analysis of osteogenesis‐related gene expression (*OPN*, *OCN*, and *RUNX2*) in BMSCs. F) RT‐qPCR analysis of chondrogenesis‐related gene expression (*COL II*, *SOX9*, and *ACAN*) in BMSCs. G) RT‐qPCR analysis of tenogenesis‐related gene expression (*COL I*, *TNMD*, and *SCX*) in TDSCs. H–J) Immunofluorescence analysis of related protein expression (OPN, COL II, and TNMD). K–M) Semi‐quantitative analysis of protein expression from immunofluorescence assays. *n* = 3. *p < 0.05, **p < 0.01, ***p < 0.001.

A Transwell chamber was used to co‐culture BMSCs with different hydrogels to compare cell proliferation. Based on live/dead cell staining and CCK‐8 results, no significant differences were observed between the groups on the first day. On days 3 and 5, the OD values were significantly higher in the HMs/MBs/ADSCs group than in the control group, with almost no dead cells and a significantly high cell count in the area (Figure S21A,B, Supporting Information). Scratch assays and Transwell migration experiments were conducted to evaluate the migration capacity of the hydrogels (Figure S22, Supporting Information). The scratch assay results demonstrated that the HMs/MBs/ADSCs group exhibited greater recovery areas (**Figure** **10**A,C), whereas crystal violet staining revealed that the HMs/MBs/ADSCs group had the highest number of migrating cells (Figure 10B,D). RT‐qPCR analysis demonstrated enhanced expression of osteogenic genes (*OPN*, *OCN*, and *RUNX2*) (Figure 10E), chondrogenic genes (*COL II*, *SOX9*, and *ACAN*) (Figure 10F), and tenogenic genes (*COL I*, *TNMD*, and *SCX*) (Figure 10G) in the HMs/MBs/ADSCs group. Immunofluorescence analysis confirmed the upregulated expression of OPN, COL II, and TNMD in the HMs/MBs/ADSCs group (Figure 10H–M).

## 3 Discussion

Tissue‐engineered hydrogels represent a promising solution for TBI treatment, as they inherently integrate ECM, stem cells, and osteochondral‐inducing components. Moreover, hydrogels can be injected into irregular TBI defect sites using minimally invasive strategies. Macroporous hydrogels have garnered increasing attention for use in tissue regeneration owing to their superior structural properties.^[^ ^27^ ^]^ In this study, the thermosensitive properties of GMs were exploited to create micropores in a hydrogel, thereby constructing a 3D porous hydrogel system. Based on previous research by the group,^[^ ^28^ ^]^ the thickness of the tissue layer formed by cell growth is ≈50–100 µm. Accordingly, the diameter of the microspheres was designed to be 1.5 or 2 times this thickness to ensure that the internal space of the microspheres was mostly filled after cell growth. When microspheres are filled with cellular tissue, adjacent microspheres may connect and eventually form a complete tissue structure. Therefore, GMs with a dry‐state diameter of 100 to 150 µm and hydrated‐state diameter of 150 to 200 µm were constructed to provide a 3D spatial interface, promoting cell growth and tissue formation within the microspheres. The hydrogel exhibited a high swelling ratio, which further increased with the addition of GMs. From the perspective of polymer swelling, the HAMA hydrogel used in this study contained numerous hydrophilic groups. In addition, the incorporation of GMs created more porous structures within the system. These pores provided additional channels for water molecules to penetrate and significantly enhanced the swelling process. This mechanism ensures that the cells within the hydrogel are supplied with sufficient water and nutrients, thereby maintaining normal cellular metabolism. As shown in Figure 3B–D, ADSCs encapsulated in porous HAMA exhibited significantly enhanced proliferation over time compared with those in non‐porous HAMA, with a tendency to aggregate. This finding indicated that the porous platform facilitated cell homing and enhanced cell proliferation. Encapsulating chondrocytes in porous GelMA hydrogels facilitates chondrocyte proliferation and promotes the secretion of ECM components, including type II collagen, type I collagen, and fibronectin, after 28 days.^[^ ^29^ ^]^ Similarly, Chang et al. utilized GMs as porogens to fabricate a 3D porous sodium alginate hydrogel that enhanced the proliferation and protein secretion of cancer cells.^[^ ^30^ ^]^

To further regulate cellular functions and sustain paracrine activity, BMP‐2 was incorporated into a porous platform. The effective release of BMP‐2 is crucial for tissue repair. Current studies indicate that the covalent attachment of growth factors to carriers is commonly accomplished through chemical reactions involving carboxyl, amine, or cysteine functional groups, facilitating long‐term release.^[^ ^31^ ^]^ However, chemical conjugation often compromises the biological activity of growth factors. From a clinical translation perspective, scaffolds preloaded with specific growth factors are difficult to preserve and exhibit limited applicability. The gentle adsorption‐based loading of BMP‐2 after drying effectively preserved its biological activity without disrupting its complex and ordered spatial structure. Most studies have performed cross‐linking prior to immersion in a growth factor or incorporated it during preparation, often resulting in significant losses.^[^ ^32^ ^]^ As the swelling rate of the microspheres before crosslinking was higher than that after crosslinking (Figure 2C), more factors could potentially be encapsulated by immersing them in the BMP‐2 solution before crosslinking. These results demonstrated that the sustained‐release effect was superior (Figure 2E). Moreover, more BMP‐2 was retained after GMM lysis, suggesting that this loading method preserved the biological activity of BMP‐2. This approach likely acts as a protective shield for simply adsorbed BMP‐2, reducing the initial burst release rate, whereas BMP‐2 adsorbed via electrostatic interactions is released only during GMMs degradation. Subsequently, the BMP‐2‐loaded microspheres were encapsulated within the porous HAMA hydrogel, providing dual protection for BMP‐2 and promoting its controlled release (Figure 2I). Compared with observations in most studies, the overall release rate of BMP‐2 was slower, with a relatively lower initial burst release rate.^[^ ^21^, ^33^ ^]^ This release profile aligns better with the requirements of the tissue repair process. The presence of BMP‐2 enabled ADSCs to establish a multifunctional interaction platform within a 3D porous environment. ADSCs encapsulated in BMP‐2 exhibited a faster proliferation rate. Additionally, the GelMA microspheres attracted ADSCs owing to their BMP‐2 activity, leading to cell adhesion on the microspheres. BMP‐2 interacts closely with the ECM. By binding to integrins on the cell surface, BMP‐2 facilitates cell adhesion and migration and exerts significant biological functions.^[^ ^34^ ^]^

Among the four‐layer structures of the TBI, fibrocartilage tissue exhibits limited self‐repair capacity and is prone to scar tissue formation, which increases the risk of re‐tearing. This has consistently been the focus of this study. Wang et al. demonstrated that BMP‐2 facilitates the repair of osteochondral tissue at the TBI.^[^ ^35^ ^]^ Lee et al. injected a collagen gel containing recombinant human BMP‐2 into the tendon–bone tunnel interface, resulting in fibrocartilage and new bone formation after 6 weeks.^[^ ^36^ ^]^ Similarly, our tissue repair outcomes demonstrated that BMP‐2 was more effective in promoting TBI healing in the rat rotator cuff injury model than in the suture‐only group. Micro‐CT results showed that the two BMP‐2 groups exhibited greater new bone formation, highlighting the potent osteoinductive capacity of BMP‐2. Histological analysis revealed that the BMP‐2 group achieved interface fusion by 6 weeks, with significant fibrocartilage formation evident at 12 weeks. Additionally, combined picrosirius red staining and immunohistochemistry of COL I and COL II confirmed that the interface in these groups formed fibrocartilage primarily composed of COL I and COL II, rather than scar tissue dominated by COL III. Additionally, TBI biomechanics demonstrated better mechanical strength. In conclusion, BMP‐2 signaling plays a critical role in the reconstruction of the fibrocartilage transition zone. However, the combination of BMP‐2 and ADSCs demonstrated superior TBI healing outcomes, including enhanced bone regeneration, mechanical strength, and interface fusion.

To explore the repair mechanism, we sequenced the samples 6 weeks after repair. Interestingly, it was discovered that in both BMP‐2‐loaded groups, the pathway promoting tissue repair was the TGF‐β/Smad2/3 pathway, rather than the classical Smad1/5/8 pathway frequently reported in the literature.^[^ ^37^ ^]^ The TGF‐β/Smad superfamily plays a pivotal role in cartilage regeneration. TGF‐β/Smad signaling is essential for cartilage generation and ECM maintenance, with multiple regulatory branches. Among them, the TGF‐β/Smad2/3 signaling pathway promotes cartilage formation while inhibiting chondrocyte hypertrophy.^[^ ^38^ ^]^ The Smad1/5/8 pathway promotes osteogenesis and inhibits ectopic ossification but induces chondrocyte hypertrophy.^[^ ^39^ ^]^ Hu et al. demonstrated through transcriptomic and proteomic analyses that TGF‐β/Smad1/5/8 inhibits osteochondral tissue regeneration.^[^ ^40^ ^]^ Zhang et al. also reported crosstalk between the TGF‐β/Smad and integrin signaling pathways, which regulate mesenchymal stem cell chondrogenesis.^[^ ^41^ ^]^ Specifically, blocking the TGF‐β/Activin/Nodal signaling pathway induces hypertrophy and activates the BMP/Smad1/5/8 and integrin signaling pathways. By contrast, inhibiting the integrin–ECM interaction suppresses hypertrophy while activating TGF‐β/Smad2/3. Thus, under different conditions, Smad2/3 and Smad1/5/8 transition between pathways to support cartilage formation. BMP‐2 can upregulate or downregulate Smad2/3 under certain conditions.^[^ ^42^ ^]^ However, no related studies have been conducted on bone tissue engineering. Chen et al. investigated cartilage regeneration mechanisms in osteoarthritis using hyaluronic acid and platelet‐rich plasma, finding that they activate TGF‐βRII and downstream Smad2/3 signaling.^[^ ^43^ ^]^ Therefore, it is hypothesized that the study mediated by porous HAMA hydrogels could stimulate fibrocartilage regeneration by BMP‐2 mainly through the TGF‐β/Smad2/3 pathway. Additionally, sequencing results revealed that TGF‐β3 was the predominant isoform expressed in the TGF‐β family, with significant release detected in the supernatants of HMs/MBs/ADSCs and BMSCs co‐culture (Figure 8I). These findings demonstrate that ADSCs exert their effects through paracrine signaling, whereas BMP‐2 enhances their activity, jointly inducing robust expression of the TGF‐β/Smad2/3 pathway. TGF‐β3, expressed in both cartilage and tendon, activates downstream signaling pathways by binding to TGF‐β receptors on the cell membrane.^[^ ^44^ ^]^ Upon receptor binding, kinase activity is activated, leading to phosphorylation of Smad2 and Smad3. Phosphorylated Smad2 and Smad3 bind to Smad4 to form a complex that translocates into the nucleus to function as a transcription factor regulating target gene expression (Figure 1C). TGF‐β3 and Smad2/3 are pivotal in tissue reconstruction.^[^ ^45^ ^]^ They promote fibroblast proliferation and collagen synthesis, thereby facilitating tissue repair. Activating the TGF‐β/Smad2/3 pathway promotes the expression of cartilage‐ and tendon‐related genes.^[^ ^46^ ^]^

In vitro experiments demonstrated that ADSCs within the 3D porous hydrogel supported by BMP‐2 not only promoted BMSCs proliferation and migration but also enhanced osteogenic and chondrogenic gene expression. Additionally, tendon‐related gene expression was upregulated in TDSCs (Figure 10G,J). BMP‐2 enhances matrix metalloproteinase expression, facilitating cell–matrix interactions and strengthening migratory capacity.^[^ ^47^ ^]^ For example, in vitro experiments showed that MSCs treated with BMP‐2 exhibited enhanced migratory activity, potentially providing additional cellular sources for tissue regeneration.^[^ ^48^ ^]^ Thus, loading BMP‐2 into the porous hydrogel may activate the TGF‐β pathway, stimulating endogenous stem cells to migrate inward and exert biological functions. BMP‐2 synergizing with ADSCs simultaneously leverages the activities of both exogenous and endogenous stem cells, leading to a more pronounced pathway activation. To investigate the mechanisms underlying ADSCs‐mediated pathway activation, we performed in vitro sequencing. The AMPK pathway plays a key role in this process, as its activation reverses mitochondrial damage caused by ROS, reduces ROS levels, and exerts antioxidant effects.^[^ ^49^ ^]^ Interestingly, the AMPK pathway was also detected during the 6‐week repair stage. Its expression indicates healthy mitochondria, ensuring adequate energy supply for tissue repair.^[^ ^50^ ^]^ Zhang et al. investigated ADSCs‐derived exosomes in preventing degenerative changes in torn human rotator cuff tendons, which may be mediated by AMPK activation, reduced proinflammatory cytokine synthesis, and increased collagen synthesis.^[^ ^25a^ ^]^ Chen et al. synthesized a biomembrane that regulates mitochondrial dynamics in dysfunctional MSCs via the AMPK pathway, reducing ROS levels and promoting bone integration at the implant interface.^[^ ^51^ ^]^ Additionally, Hu et al. demonstrated that APN, a fat‐derived adipokine, reverses mitochondrial dysfunction and improves titanium implant integration under diabetic conditions via the AMPK pathway.^[^ ^52^ ^]^ APN possesses strong antioxidant, mitochondrial protective, and anti‐diabetic effects. AMPK phosphorylation is a key mechanism of APN activity, with most studies focusing on the cardiovascular and endocrine systems.^[^ ^53^ ^]^ APN binds to its receptors (AdipoR1 and AdipoR2) to initiate intracellular signaling. Interestingly, sequencing revealed significant APN upregulation in HMs/MBs/ADSCs, and ELISA detected high APN concentrations (Figure 8H). Therefore, in the 3D porous hydrogel system, ADSCs primarily affect local tissues via paracrine secretion, whereas BMP‐2 enhances their paracrine activity, consistent with previous findings.^[^ ^19^ ^]^ Moreover, BMP‐2 amplified ADSC paracrine activity and significantly increased phosphorylated AMPK expression. Sequencing results also revealed the involvement of fatty acid metabolism, a critical pathway for cellular energy production. In tissue injury microenvironments, β‐oxidation generates substantial ATP to meet the high energy demands of repair and proliferation.^[^ ^54^ ^]^ The differentiation potential and paracrine effects of exogenous ADSCs may indirectly influence fatty acid synthesis.^[^ ^55^ ^]^ Their paracrine effects involve growth factors and cytokines that regulate fatty acid synthesis gene expression. This study demonstrated that ADSCs activate the AMPK pathway via APN secretion, further influencing fatty acid metabolism.^[^ ^56^ ^]^ These mechanisms collectively promote tissue regeneration and functional recovery.

In summary, this study successfully achieved multistage synergistic regulation of TBI repair by constructing a 3D porous HAMA hydrogel system loaded with BMP‐2. At the material design level (Figure 1A), thermosensitive GMs degraded at body temperature, forming a microporous network that provided space for ADSC proliferation. Simultaneously, the controlled release properties of GMMs ensured sustained BMP‐2 release and guided cell migration, leading to the gradual aggregation of ADSCs around the pores and GMMs. The in vivo repair process (Figure 1B,C) demonstrated a dual protective mechanism. During the early oxidative stress phase, BMP‐2 stimulated ADSCs to activate the AMPK pathway via APN paracrine secretion, clearing ROS and restoring mitochondrial function in BMSCs, thereby preserving their biological activity. In the later repair phase, BMP‐2 not only promoted osteogenic differentiation but also synergized with TGF‐β3 secreted by ADSCs to activate the TGF‐β/Smad2/3 pathway, inducing BMSC differentiation into chondrocytes and fibrocartilage formation, as well as promoting tendon regeneration by TDSCs. This process ultimately reconstructed the three‐layer structure of bone, cartilage, and tendon. By integrating material design with biological regulation, this composite hydrogel system provides an innovative therapeutic strategy for TBI repair.

Our current study has certain limitations. First, the acute rotator cuff injury model used in this research differs from the more clinically common chronic injury model. Additionally, the hydrogel properties were designed primarily for an open surgical environment, while arthroscopic surgery is more prevalent in clinical practice. Future studies should optimize hydrogel characteristics for the specific conditions of arthroscopic surgery. Second, although the ideal source of ADSCs should be autologous transplantation, allogeneic ADSCs were used in this study due to considerations of resource efficiency and the relatively mild immune response in rats. The biocompatibility of these cells was confirmed through in vivo biosafety and histological evaluations. Finally, since in vitro models cannot fully replicate the in vivo environment, a dual‐sequencing approach (in vivo and in vitro) was adopted to explore the relevant mechanisms. While these mechanisms were validated both in vivo and in vitro, further verification through knockout of key genes in the related pathways could enhance the robustness of the conclusions.

## 4 Conclusion

This study developed an injectable porous hydrogel‐based cell‐loaded construct (HMs/MBs/ADSCs). GMMs encapsulated BMP‐2 using a unique method to achieve sustained release. Simultaneously, a porous HAMA hydrogel system was constructed using GMs, providing a 3D bioactive environment for ADSCs and promoting ADSCs proliferation and paracrine effects. This construct significantly enhanced TBI healing and fibrocartilage transition zone reconstruction in a rat rotator cuff injury model. Both in vivo and in vitro analyses demonstrated the critical roles of the AMPK and TGF‐β/Smad2/3 signaling pathways in TBI repair. Mechanistic studies showed that HMs/MBs/ADSCs leveraged paracrine effects to clear ROS and protect mitochondria via the AMPK pathway in the early stages, supplying energy for tissue repair. In the later stages, the TGF‐β/Smad2/3 pathway was activated to promote fibrocartilage and tendon regeneration. By integrating material design with mechanistic exploration, this study not only enhances the application potential of tissue‐engineered hydrogels but also provides new insights for clinical treatment.

## Conflict of Interest

The authors declare no conflict of interest.

## Supporting information



Supporting Information

## Data Availability

The data that support the findings of this study are available from the corresponding author upon reasonable request.
