# Supplementary material for: An Injectable BMP‐2‐Releasing Porous Hydrogel Regulating the Paracrine Effects of ADSCs Promotes Tendon‐to‐Bone Healing in Rotator Cuff Repair
Source: Adv Sci (Weinh). 2025 Jun 23;12(35):e15923. doi: 10.1002/advs.202415923 (PMC12462992; doi:10.1002/advs.202415923)
Supplement: Supplementary file 1 — Supporting Information [file ADVS-12-e15923-s001.docx]

**Supporting Information**

**An Injectable** **BMP-2-Releasing Porous Hydrogel** **Regulating the Paracrine Effects of ADSCs Promotes Tendon-to-Bone Healing in Rotator Cuff Repair**

*Meijuan Yuan ^a,b,c,1^, Xiaomei Dai ^b,c,1^, Yuxia Yang ^b,c,d,1^, Shuai Liu ^c,e^, Ziyi Xu ^a,b,c^, Liang Wang ^c,d^, Junli Shi ^f^, Wenkang Liu ^c,d^, Jian Yang ^c,d^, Dianwei Liu ^c,e^, Hang Yao ^b,f,*^, Wenyong Fei ^b,c,*^*

^a^ School of Nursing and School of Public Health, Yangzhou University, Yangzhou 225001, P. R. China

^b^ Center of Basic and Clinical Research in Sports Medicine, Yangzhou University, Yangzhou, Jiangsu 225001, P. R. China

^c^ Department of Orthopedics and Sports Medicine, Northern Jiangsu People's Hospital, Affiliated to Yangzhou University, Yangzhou 225001, P. R. China

^d^ Medical College, Yangzhou University, Yangzhou 225001, P. R. China

^e^ Department of Orthopedics and Sports Medicine, The Yangzhou school of clinical medicine of Dalian Medical University, Dalian 116044, P. R. China

^f^ School of Chemistry and Chemical Engineering, Yangzhou University, Yangzhou 225009, P. R. China

^1^ The authors contributed equally to this work.

*Co-Corresponding Authors:

Hang YAO, Ph.D.

School of Chemistry and Chemical Engineering

Yangzhou University

Yangzhou 225009, P. R. China

Email: [yaohang@yzu.edu.cn](mailto:yaohang@yzu.edu.cn)

Wenyong FEI, Ph.D.

Department of Orthopedics and Sports Medicine, Northern Jiangsu People's Hospital

Affiliated to Yangzhou University

Yangzhou 225001, P. R. China

Email: sbydyx105@163.com

**1. Experimental Section**

**1.1. Preparation of microspheres**

Based on previous studies,^[1]^ the oil-in-water and oil-in-oil (oil/water/oil) double-emulsion principles were used to prepare hollow microspheres. Briefly, 10 mL of ethyl acetate was mixed with 25 mL of a 10% gelatin (Sigma-Aldrich, USA)/ gelatin methacryloyl (GelMA) solution synthesized using previously described methods to form an emulsion.^[2]^ This mixture was then combined with soybean oil and gelated on ice. The filtered pellets were dispersed in precooled ethanol, washed three times with dioxane, acetone, and anhydrous ethanol, and dried overnight to obtain dry gelatin microspheres (GMs) and uncrosslinked gelatin methacryloyl hydrogel microspheres (GMMs). Both types of microspheres were screened to 100–150 μm. Uncrosslinked GMMs were dispersed in precooled 0.5% 2-hydroxy-4'-(2-hydroxyethoxy)-2-methylpropiophenone (photo-initiator, I2959, Sigma-Aldrich, USA) and irradiated with UV for 3 min. The microspheres were then filtered, washed, and dried to obtain crosslinked GMMs. The dried microspheres were placed in an oven at 120 °C for 2 h, rapidly cooled at –20 °C for 0.5 h, and then dispersed in precooled 10% penicillin–streptomycin solution for 24 h. The microspheres were stored in phosphate-buffered saline (PBS) at 4 °C for later use.

**1.2. Loading of BMP-2 and preparation of composite hydrogels**

Recombinant human bone morphogenetic protein-2 (rhBMP-2) was obtained from Jinyuan Gene Engineering (China). BMP-2 was loaded onto GMMs at a concentration of 1 μg/mL using two methods: GMBs1: Dry, crosslinked GMMs were immersed in BMP-2 solution (1 μg/mL) at 4 °C overnight for full swelling; GMBs2: Dry, uncrosslinked GMMs were immersed in BMP-2 solution (1 μg/mL) at 4 °C overnight for full swelling and then dispersed in 0.5% I2959 solution and irradiated with UV for 3 min. HAMA hydrogel was synthesized using a previously described method.^[3]^ A 2% HAMA solution was prepared in 0.5% I2959 solution, and GMs as well as GMBs were mixed into the HAMA solution at a wet weight of 0.3 g/mL to obtain a composite hydrogel precursor. The mixture was injected into a mold and irradiated with UV for 3 min to form the HMs/MBs hydrogel. Similarly, GMs were uniformly added to 2% HAMA solution at a wet weight of 0.3 g/mL, followed by UV curing to form HMs hydrogel. Additionally, 1 μg/mL of BMP-2 solution was directly mixed into the HAMA prepolymer, followed by uniform blending and UV cross-linking to form HB hydrogel. GMBs (wet weight: 0.3 g/mL) were also incorporated into the HAMA prepolymer and UV cross-linked to form HMBs hydrogel.

**1.3. Characterization of the** **microspheres and composite hydrogels**

The synthesis of GelMA and HAMA was validated using ¹H NMR spectrometry (400 MHz, Agilent, USA) and Fourier-transform infrared spectroscopy (Thermo Fisher Scientific, USA).

SEM (ZEISS, Germany) was used to observe the morphology of the dried microspheres, whereas wet microspheres were examined under a microscope (Olympus, Tokyo, Japan). The cross-section of the composite hydrogel was analyzed using SEM after swelling at 37 °C for 24 h, followed by freeze-drying.

The mechanical behavior of the hydrogel samples was evaluated through rheological analysis, employing a rotational rheometer (MARS 60, Thermo Fisher Scientific, United States). To characterize the flow properties of the pre-gel solutions, the shear rate was systematically raised from 0.1 to 100 s⁻¹. Additionally, the storage and loss modulus (G′ and G″) were monitored during a 3-minute interval under ultraviolet light exposure. For crosslinked hydrogel blocks (8 mm in diameter; 1 mm thick), G′ and G″ were analyzed under strains ranging from 0.1% to 100% at a fixed frequency of 1 Hz, while their values were also recorded over a frequency range of 0.1 to 10 rad/s at a constant strain of 1%. Additionally, axial compression tests were performed on the crosslinked hydrogels at a rate of 1 mm/min, and the compression modulus was calculated by fitting the initial 15% strain segment of the stress–strain curve.

For the swelling test of the microspheres and hydrogel blocks (6 mm in diameter; 1 mm thick), the initial dry weight of the sample (W₀) was measured before immersion in deionized water. The sample was then weighed (Wₜ) after removing excess water at specific time points. Microspheres were placed at 4 °C to swell for 24 h before collection. Hydrogel blocks (8 mm in diameter; 1 mm thick) were placed on a shaker at 37 °C, 80 rpm, and collected at 30, 60, 90, 120, 240, and 360 min. The swelling rate was calculated as: swelling rate (%) = ((Wₜ – W₀) / W₀) × 100%. The swelling ratio was used to indirectly assess the degree of crosslinking. The swelling ratio was calculated as: swelling ratio = Wₜ / W₀.

For the microsphere degradation test, 20 mg of dry GMs and GMMs were fully swollen in PBS and placed in small dishes. The number of microspheres was counted using a microscope (T₀). The samples were incubated at 37 °C, 80 rpm, with the PBS solution replaced every 3 d. For GMs, degradation was assessed at 10, 20, 30, and 40 min, whereas for GMMs, time points were set at 1, 3, 5, 7, 9, 14, 21, and 28 d. The microspheres were counted under a microscope at each time point (T₁), and the degradation rate was calculated using: degradation rate (%) = ((T₁ – T₀) / T₀) × 100%.

For the *in vitro* degradation test of the hydrogel blocks (8 mm in diameter; 1 mm thick), the initial dry weight (W₀) was recorded. Samples were then immersed in 1 mL PBS and incubated on a shaker at 37 °C, 100 rpm, with PBS replaced every 3 d. At predetermined time points, the samples were removed, rinsed with deionized water to eliminate salts, freeze-dried, and weighed (Wₑ). The degradation rate was determined using: degradation rate (%) = ((W₀ – Wₑ) / W₀) × 100%.

**1.4. ELISA-based detection of BMP-2 release from GMBs1, GMBs2, and different hydrogel blocks**

GMMs (30 mg dry weight) were fully loaded with BMP-2 and immersed in 1.2 mL PBS, followed by incubation on a shaker (37 °C, 80 rpm). At specific time points, a 1 mL aliquot of the supernatant was carefully transferred and preserved at a temperature of –80 °C. Subsequently, the same quantity of PBS was introduced to replenish the volume. The released BMP-2 in the supernatant was analyzed using a BMP-2 ELISA kit (Thermo Fisher Scientific), and the cumulative release rate was determined. On day 28, GMBs were lysed using 0.3 mg/mL GelMA lysis buffer (EFL, China), and the BMP-2 concentration following complete lysis was measured. Following a similar procedure, The hydrogel constructs, including HB, HMBs, and HMs/MBs (prepared as described in Section 1.2), were submerged in PBS. BMP-2 release was quantified using a BMP-2 ELISA kit. The cumulative release rate of BMP-2 was calculated as: cumulative release rate (%) = (Mₙ / M₀) × 100%, where Mₙ denotes the cumulative BMP-2 released at time point *n*, and M₀ is the initial total BMP-2 amount.

**1.5. Cell culture**

Primary stem cells, including adipose-derived stem cells (ADSCs), bone marrow-derived stem cells (BMSCs), and tendon-derived stem cells (TDSCs) isolated from rats, were obtained from Procell (Wuhan, China). These cells were maintained in low-glucose Dulbecco’s Modified Eagle Medium (DMEM, Gibco, USA), which was enriched with 10% fetal bovine serum (FBS, Corning, USA) and 1% penicillin–streptomycin solution (PS, Beyotime, China). The cultures were incubated under controlled environmental conditions at 37 °C with 5% CO₂. All stem cells were cultured until the third passage before use.

**1.6. Biocompatibility and distribution of ADSCs in porous hydrogels *in vitro***

To compare the cytocompatibility of different HAMA concentrations, ADSCs were plated in 24-well culture plates and incubated with extracts derived from varying concentrations of HAMA. CCK-8 and live/dead staining assays (Beyotime, China) were performed on days 1, 3, and 5.

After passaging and centrifugation, third-generation ADSCs were resuspended at a uniform density of 5 × 10⁶ cells/mL in the prepared HAMA, HMs, and HMs/MBs hydrogel precursors (prepared according to Method 1.2) and thoroughly mixed. The ADSCs–hydrogel mixture (100 μL per aliquot) was injected into molds (8 mm in diameter; 1 mm thick) using a syringe, followed by UV exposure for 3 min to form HAMA/ADSCs, HMs/ADSCs, and HMs/MBs/ADSCs hydrogels. The prepared ADSCs-loaded hydrogels were cultured in complete medium in an incubator (5% CO₂; 37 °C), with medium replacement every 3 d. To assess cell proliferation within the hydrogels on days 1, 7, and 14, hydrogel blocks were ground at predetermined time points. DNA was extracted using the BeyoMag Universal Genomic DNA Purification Mini Kit with Magnetic Beads (Beyotime, China), and the DNA content was measured. Live/dead and nuclear/skeletal staining assays (Beyotime, China) were performed on days 1, 7, and 14. Encapsulated ADSCs were imaged using a confocal microscope (LSM710; Zeiss, Germany).

On day 14, ADSCs-coated hydrogels were fixed with paraformaldehyde, and uncoated hydrogels were sectioned using a freezing microtome (CM1950; Leica, Germany). Sectioned samples were stained with Masson's trichrome (Masson, Solarbio) and Safranin O (SO, Sigma, USA).

**1.7. Establishment of a rat rotator cuff injury model**

Male Sprague–Dawley rats (approximately 300 g) were obtained from the Experimental Animal Center of Yangzhou University. All procedures involving animal use, care, and handling were approved by the Animal Welfare Ethics Committee of Yangzhou University (Approval No.: 202306032). Rats were randomly assigned to three groups: Blank, HMs/MBs, and HMs/MBs/ADSCs (Figure 4A). The HMs/MBs and HMs/MBs/ADSCs hydrogel precursors were prepared according to Sections 5.4 and 5.6, respectively. A rat rotator cuff injury model was constructed based on literature and prior research.^[4]^ The surgeries were independently performed by a clinician with proficient surgical skills and extensive anatomical knowledge to minimize variability in surgical operations. The procedure was as follows: Isoflurane was used for continuous inhalation anesthesia. Anesthetized rats were fixed to fully expose the surgical area of the shoulder. A longitudinal incision was made, and the muscles, fascia, and other tissues were carefully separated using blunt dissection. The supraspinatus tendon was identified and lifted using a needle (Figure S9A). The supraspinatus tendon was cut with a surgical scalpel and separated from the humeral head. Residual tendon tissue at the interface was carefully scraped away using a surgical blade, leaving the surface of the humeral head completely exposed (Figure S9B). For the HMs/MBs and HMs/MBs/ADSCs groups, 50 μL of their hydrogel precursors was injected at the interface (Figure S9C), followed by UV exposure for 3 min for curing. After solidification, the tendon was sutured to the humerus (Figure S9D). Finally, the muscle and skin were sutured layer-by-layer to complete the procedure. The blank control group underwent direct suturing. At 6 and 12 weeks post-surgery, the rat supraspinatus–humerus complex was harvested for further analysis.

**1.8. *In vivo* biosafety assessment**

Two weeks after rotator cuff repair surgery, rats were fasted for 12 h, after which blood and major organs (heart, liver, spleen, lungs, and kidneys) were collected (n = 3). Blood samples were used for routine hematological and biochemical tests, including white blood cell count (WBC), red blood cell count (RBC), hemoglobin level (HGB), platelet count (PLT), alanine aminotransferase (ALT), aspartate aminotransferase (AST), urea nitrogen (UREA), uric acid (UA), and creatinine (CREA). Organs were preserved in paraformaldehyde, followed by dehydration, paraffin embedding, and H&E staining for histological examination.

**1.9. *In vivo* degradation of different hydrogels**

Hydrogel blocks (8 mm in diameter; 1 mm thick) were subcutaneously implanted into rats for degradation testing (n = 3). The degradation of the hydrogels was observed at 2 and 4 weeks.

**1.10. Survival of ADSCs *in vivo***

Third-passage ADSCs were cultured in dishes until they reached 80-90% confluence. They were then labeled with DIR, a lipophilic membrane-labeling dye, and incubated in a 5 μM DiR solution (Yeasen, China) for one hour at 37 °C. The ADSCs were then washed three times and cultured in complete medium for 2 d. Finally, ADSCs were resuspended in the HMs/MBs hydrogel precursor at a density of 5 × 10⁶ cells/mL. A 50 μL volume of DiR-labeled HMs/MBs/ADSCs hydrogel precursor was injected into the TBI of the rat rotator cuff injury model as described previously, followed by UV curing. The average number of cells was 2.5 × 10⁵. Cell survival was monitored using a small-animal *in vivo* imaging system (*In vivo* Xtreme II; Bruker, Germany). Images were analyzed using Bruker MI SE. Data acquisition was conducted at predetermined time intervals, specifically on days 0, 7, 14, 21, 28, 35, and 42. The fluorescence detection parameters were configured with an excitation wavelength of 748 nm and an emission wavelength of 780 nm.

**1.11. *In vivo* inflammation assessment by immunofluorescence**

At 2 weeks post-rotator cuff repair surgery, the tendon–bone complex of the rotator cuff was collected, fixed in paraformaldehyde, decalcified, dehydrated, and embedded in paraffin for sectioning. For immunofluorescence staining, the sections were deparaffinized and incubated overnight with primary antibodies against CD68 and CD163. After washing with PBS, the sections were incubated with corresponding secondary antibodies. Cell nuclei were stained with DAPI for visualization. The prepared specimens were subsequently examined using a fluorescence microscope (Olympus, Japan). Quantitative assessment of fluorescence signal intensity was carried out utilizing ImageJ analysis software.

**1.12. Micro-CT analysis and biomechanical testing**

The supraspinatus–humerus complex (n = 3) was scanned using a micro-CT system (SkyScan 1176, Bruker, Germany) at 12 weeks postoperatively. The images were analyzed using CTAn software, which was used to analyze the footprint of supraspinatus tendon insertion. The analyzed parameters included BMD, BV/TV fraction, Tb.N, and Tb.Sp.

Specimens (n = 5) were collected at 6 and 12 weeks, and the transverse area of the middle supraspinatus tendon was measured using digital Vernier calipers. The biomechanical strength of the supraspinatus–tendon–bone complex was measured using a modified rheometer. The complex was pre-stretched at 0.1 N and then loaded at a fixed speed of 10 mm/min until failure. The ultimate failure load was measured, and the stiffness was determined based on the load–displacement curve. The stress was calculated using the formula: Stress = Ultimate load to failure / Cross-sectional area.

**1.13. Histological and immunohistochemical analyses**

At 6 and 12 weeks post-rotator cuff repair surgery, tendon–bone complexes were collected, fixed, and decalcified before sectioning. After embedding in paraffin, histological and immunohistochemical sections were prepared. Tissue sections were stained with H&E, Masson's trichrome, Safranin O-fast green, and picrosirius red. Histological scoring was performed as previously described (**Table S1**). ImageJ software was used for the semi-quantitative analysis of the areas positive for Safranin O-fast green staining. Picrosirius red images were acquired using a polarized light microscope (Olympus, Tokyo, Japan) to evaluate COL I/COL III content and collagen fiber organization. COL I was identified as yellow or red, whereas COL III was identified as green.^[5]^ Semi-quantitative analysis of each positive area was performed using ImageJ software. Additionally, immunohistochemical staining for COL I and COL II was performed, and positively stained areas were semi-quantitatively analyzed using ImageJ software. The antibodies used for immunohistochemistry are listed in **Table S2**.

**1.14. *In vivo* RNA sequencing of the tendon–bone complex and *in vitro* RNA sequencing of HMs/ADSCs and HMs/MBs/ADSCs hydrogels**

At the 6-week time point, the supraspinatus tendon–bone interface tissues were collected from all experimental groups and rapidly frozen using liquid nitrogen for preservation. All tissue samples were collected, stored, and processed in the same batch. RNA extraction and sequencing were performed in a single batch, and all samples were processed simultaneously for library preparation and sequencing. The batch effects between samples were negligible. RNA extraction, library construction, sequencing, and analysis were conducted by Shanghai Genechem Co. Ltd. Gene expression with a fold change exceeding 2 and a p-value below 0.05 was considered significant. Gene Ontology and KEGG analyses were conducted to identify enriched biological processes and pathways associated with the genes of interest.

*In vitro*, ADSCs were encapsulated within the porous hydrogel and divided into HMs/ADSCs and HMs/MBs/ADSCs groups (Method 5.6). After 14 d of culture, the ADSCs-loaded hydrogel constructs were collected. RNA extraction, library construction, sequencing, and analysis were conducted by BGI Tech Solutions Co., Ltd. This analysis was performed as previously described.

**1.15. Validation by immunofluorescence and western blot of *in vivo* RNA sequencing**

Following the aforementioned methods, immunofluorescence and western blot analyses were performed on 6-week sections and tendon–bone complexes, respectively. Immunofluorescence analysis was conducted to detect p-Smad2/3 and p-AMPK levels. The expression of TGF-β/Smad2/3 and AMPK pathway-related proteins (Smad2/3, p-Smad2/3, AMPK, and p-AMPK) was detected by western blotting. The antibodies used for western blot and immunofluorescence analyses are listed in Table S2. The relative optical densities of protein bands and fluorescence signal intensities were quantitatively analyzed using ImageJ software.

**1.16. Construction of an oxidative stress model *in vitro***

To simulate an oxidative stress microenvironment, BMSCs were seeded into 24- or 6-well plates and cultured until 70% confluency was reached. The cells were then treated with 500 μM H₂O₂ in serum-free medium for 24 h, designated as the H₂O₂ group.^[6]^ Under these conditions, the survival status of BMSCs was evaluated, with the experiment divided into a control group and an H₂O₂ group. The control group was cultured in serum-free medium for 24 h. After 24 h, both groups were switched to complete culture medium and cultured for an additional day. Cell viability and proliferation were evaluated using the CCK-8 assay and live/dead staining at three time points: before, immediately after, and 1 d after the intervention.

**1.17. Related immunofluorescence and WB validation in *in vitro* RNA sequencing under oxidative stress model**

Based on the aforementioned *in vitro* model construction method, BMSCs were divided into four groups: Control, H₂O₂, H₂O₂+HMs/ADSCs, and H₂O₂+HMs/MBs/ADSCs. The control group and H₂O₂ group were not treated. Transwell chambers (8 μm; Nest, China) containing HMs/ADSCs or HMs/MBs/ADSCs were placed above the BMSCs and co-cultured for 72 h to characterize antioxidant effects and validate the AMPK pathway. Subsequently, an additional 14 d of co-culture was conducted to validate the TGF-β/Smad2/3 pathway (Figure 8A).

BMSCs in 24-well plates were fixed with paraformaldehyde for immunofluorescence detection of p-AMPK and p-Smad2/3. BMSCs in 6-well plates were digested, centrifuged, and processed for protein extraction, followed by western blot analysis for AMPK, p-AMPK, Smad2/3, and p-Smad2/3.

**1.18. Paracrine activity and antioxidant activity of** **ADSCs-loaded hydrogel constructs under an oxidative stress model *in vitro***

The supernatants from the four groups (Control, H₂O₂, H₂O₂+HMs/ADSCs, and H₂O₂+HMs/MBs/ADSCs) were collected after 3 d of co-culture, and the concentrations of APN, TNF-α, and IL-1β were quantified using ELISA kits (Solarbio, China). Free radical-scavenging capacity was assessed using DPPH and ABTS assays. For the DPPH assay, DPPH was premixed with methanol at a 1:1 ratio and then combined with the supernatants from the four groups. The reaction mixture was incubated for 20 minutes at room temperature, after which the optical density at a wavelength of 515 nm was determined using a microplate reader (TECAN, Switzerland). For the ABTS assay, an ABTS working solution was prepared in advance and mixed with the supernatants from the four groups. The reaction was allowed to proceed for 4 min, and the absorbance at 734 nm was measured using a microplate reader. The free radical scavenging rate (%) was calculated as follows: scavenging rate = (*A_o_* – *A_1_*) / *A_o_* × 100%, where A₀ refers to the blank absorbance value, and A_1_ refers to the sample absorbance value. Supernatants collected after 14 d of co-culture were used for TGF-β ELISA detection (BOSTER, China).

ROS and superoxide levels in BMSCs were assessed using an ROS Assay Kit (Beyotime, China) and MitoSox Mitochondrial Superoxide Indicator (Thermo Scientific, USA), following the manufacturer’s protocols. Mitochondrial membrane potential (Δψm) was assessed using a JC-1-based Δψm assay kit (MCE, USA). Nuclei were subsequently stained with Hoechst 33258 (Beyotime). The ATP concentration in BMSCs was measured using an ATP assay kit (Beyotime, China), and luminescence values were recorded using a multifunctional enzyme marker (TECAN, Switzerland). The intracellular protein concentration was quantified using a BCA protein assay kit (Beyotime, China), and ATP levels were normalized to the protein content.

**1.19. Effects of** **ADSC-loaded hydrogel constructs on BMSC proliferation**

All experiments were conducted in three groups: Control, HMs/ADSCs, and HMs/MBs/ADSCs, where ADSCs were pre-cultured in 3D porous hydrogels for 14 d. Transwell chambers were used for subsequent experiments. BMSCs were seeded in 24-well plates and cultured for 24 h. The control group received no treatment, whereas the ADSCs-loaded hydrogel constructs, with or without BMP-2, were positioned in the upper chamber. CCK-8 and live/dead staining assays were performed on BMSCs in the lower chamber on days 1, 3, and 5.

**1.20. Effects of ADSCs-loaded hydrogel constructs on BMSCs migration and differentiation**

A wound healing assay was conducted to evaluate cellular migration capabilities. BMSCs were plated in 6-well culture plates at a concentration of 2 × 10⁴ cells per well. After achieving 90% confluency, a linear wound was introduced into the cell monolayer using a sterile 200-μL pipette tip. The wound areas were imaged at 0, 12, and 24 hours post-scratching using an inverted microscope. To further investigate cell migration, a Transwell migration assay was employed. BMSCs were seeded into the upper compartment of Transwell inserts at a density of 1 × 10⁴ cells, while hydrogel samples were positioned in the lower chamber. Following 24 hours of incubation, non-migratory cells on the upper surface of the membrane were carefully removed with cotton swabs. The migrated cells were fixed with 4% paraformaldehyde for 20 minutes and then stained with 0.1% crystal violet solution (Solarbio, China) for visualization.Microscopic images were obtained, and cell migration was quantitatively analyzed using ImageJ software.

To simulate the *in vivo* environment, BMSCs were used to assess osteogenic and chondrogenic differentiation capacity, whereas TDSCs were used to assess tendon differentiation capacity. The cells were seeded in the lower chamber of a 6- or 24-well plate. After 24 h, the control group received no treatment, whereas hydrogels from the two experimental groups were placed in the upper chamber. After 14 d of co-culture, RT-qPCR was performed to analyze the expression of osteogenic (*OPN*, *OCN*, and *RUNX2*), chondrogenic (*COL II*, *SOX9*, and *ACAN*), and tendinogenic (*COL I*, *TNMD*, and *SCX*) genes. The primer sequences are listed in **Table S3**. The expression of related proteins (OPN, COL II, and TNMD) was detected using immunofluorescence. The antibodies used for immunofluorescence analysis are summarized in Table S2.

**1.21 Statistical analysis**

All experimental results are expressed as mean values ± standard deviation (SD). Statistical analyses and graphical representations were performed using GraphPad Prism 9. For comparisons involving two groups, an unpaired Student's t-test was utilized, whereas one-way analysis of variance (ANOVA) was employed for evaluating differences among three or more groups. Statistical significance was denoted as follows: **p* < 0.05, ***p* < 0.01, and ****p* < 0.001.

**2. Figures**

**Figure S1.** ^1^H NMR spectra of Gelatin and GelMA.

**Figure S2.** A) ^1^H NMR spectra of HA and HAMA. B) FT-IR spectra of HA and HAMA.

**Figure S3.** Comparison of the rheological and mechanical properties of different concentrations of HAMA. A)Viscosity changes in 1%, 1.5% and 2% concentrations of HAMA. B) Dynamic modulus of different hydrogels after 3-min UV irradiation. C) Dynamic modulus of 10^-1^ to 10^2^ strain at a constant frequency of 1 Hz. D) Dynamic modulus of 10^-1^ to 10 ω at a constant oscillation amplitude (γ) of 1 %. E) Stress-strain curves of HAMA in different concentrations. F) Compressive strength at 15 % strain. (n=3). **p*＜0.05, ***p*＜0.01, ****p*＜0.001.

**Figure S4.** Comparison of the compatibility of the extracts of HAMA with different concentrations on ADSCs. A) Live/dead staining of BMSCs treated with the extracts of HAMA with different concentrations. B) Effects of different extracts on BMSCs proliferation assessed by CCK-8 assay. (n=3).

**Figure S5.** Injectable properties of prepolymers and rheological and mechanical properties of hydrogels. A) Viscosity changes in HAMA, HMs, HMs/MBs. B) Dynamic modulus of different hydrogels after 3-min UV irradiation. C) Dynamic modulus of 10^-1^ to 10^2^ strain at a constant frequency of 1 Hz. D) Stress-strain curves of different hydrogels. E) Compressive strength at 15 % strain. (n=3). ***p*＜0.01.

**Figure S6.** The swelling ratio of different hydrogels in water. (n=3).

**Figure S7.** Confocal images of nuclear and cytoskeletal staining of ADSCs coated in different hydrogels.

**Figure S8.** Macropore formation and GMBs distribution in the different hydrogel. A)Safranin staining of different hydrogels. B)Masson's trichrome staining of different hydrogels. GMBs are marked with yellow arrows.

**Figure S9.** Construction of rotator cuff injury model in rats. A) Exposure of supraspinatus tendon. B) A scalpel was used to cut the supraspinatus tendon. C) HMs/MBs/ADSCs hydrogel precursor was injected. D) The supraspinatus tendon was sutured to the humerus.

**Figure S10.** Representative images of H&E staining of major organs (hearts, liver, spleen, lungs, and kidneys) explanted from rats at 2 weeks postoperation. (n=3).

**Figure S11.** Results of routine blood test and liver and kidney function tests performed for rats at 2 weeks postoperation. (n=3).

**Figure S12.** Images of degradation of HMs/MBs and HMs/MBs/ADSCs hydrogel *in vivo*. (n=3).

**Figure S13.** The IVIS images of DIR-labeled ADSCs were taken at day 0, 7, 14, 21, 28, 35, 42 in HMs/MBs/ADSCs group. (n=3).

**Figure S14.** HMs/MBs/ADSCs improved inflammatory responses. A–B) Immunofluorescence staining and semi-quantitative analysis of CD68 2 weeks after surgery. C–D) Immunofluorescence staining and semi-quantitative analysis of CD163 2 weeks after surgery. (n=3). **p*＜0.05, ** *p*＜0.01, *** *p*＜0.001.

**Figure S15.** Representative Masson's trichrome staining of TBI.

**Figure S16.** Semi-quantitative analysis of collagen composition after 6 weeks in Picrosirius red staining. **p*＜0.05, ** *p*＜0.01, *** *p*＜0.001.

**Figure S17.** Transcriptome analysis *in vivo*. A-C) Volcano map *in vivo*. D-F) Heat map *in vivo*. G) Heatmap of genes associated with cell differentiation and fat metabolism. H) GO enrichment analysis *in vivo*.

**Figure S18.** Transcriptome analysis *in vitro*. A) Volcano map *in vitro*. B) Heat map *in vitro*. C) GO enrichment analysis *in vitro*.

**Figure S19.** Survival of BMSCs stimulated by H_2_O_2_. A) The proliferation of BMSCs before and after H_2_O_2_ intervention was measured by CCK-8 assay. B) Live/dead staining of BMSCs before and after H_2_O_2_ intervention. (n=3). **p*＜0.05, ** *p*＜0.01, *** *p*＜0.001.

**Figure S20.** A) Semi-quantitative analysis of p-AMPK by immunofluorescence *in vitro*. B) Semi-quantitative analysis of p-Smad2/3 by immunofluorescence *in vitro*. **p*＜0.05, ** *p*＜0.01, *** *p*＜0.001.

**Figure S21.** HMs/MBs/ADSCs promoted BMSCs proliferation. A) Live/dead staining of BMSCs treated with different hydrogel. B) Effects of different hydrogel on BMSCs proliferation assessed by CCK-8 assay. (n=3). **p*＜0.05, ** *p*＜0.01, *** *p*＜0.001.

**Figure S22.** Cell migration experiment. A) Diagram of the scratch experiment. B) Schematic diagram of transwell migration experiment.

**Table S1.** Details of the Histological Score System utilized in this work for evaluating tendon-bone healing.

| Individual Outcomes | Score | | | |
| --- | --- | --- | --- | --- |
|  | 1 | 2 | 3 | 4 |
| Cellularity (inflammation) | Marked | Moderate | Mild | Minimal |
| Presence of fibrocytes (%) | <25 | 26-50 | 51-75 | >75 |
| Proportion of cells oriented parallel (%) | <25 | 26-50 | 51-75 | >75 |
| Proportion of fibers oriented parallel (%) | <25 | 26-50 | 51-75 | >75 |
| Presence of matrix | Minimal | Mild | Moderate | Marked |
| Insertion integrity | C(+), R(-), F(-), T(-) | C(+), R(+), F(-), T(-) | C(+), R(+), F(+), T(-) | C(+), R(+), F(+), T(+) |
| Insertion continuity (%) | < 25 | 26-50 | 51-75 | > 75 |

C: continuity; R: regularity; F: fibrocartilage; T: tidemark; +: positive.

**Table S2.** Antibodies for immunohistochemistry, immunofluorescence, and western blot.

| Name | Company | Cat# | Name | Company | Cat# |
| --- | --- | --- | --- | --- | --- |
| COL I | Servicebio | GB11022-3 | p-AMPK | GeneTex | GTX52341 |
| COL II | Servicebio | GB11021 | OPN | Proteintech | 22952-1-AP |
| GAPDH | Proteintech | 60004-1-Ig | COL II | Abcam | ab34712 |
| Smad2/3 | Cell Signaling Technology | 8685s | TNMD | Affinity | DF-13715 |
| p-Smad2/3 | Thermo | PA5-110155 | CD68 | Proteintech | 25747-1-AP |
| AMPK | Proteintech | 10929-2-AP | CD163 | Proteintech | 16646-1-AP |

**Table S3.** Sequences of primers used for qRT-PCR.

| Gene | Species | Forward Primer | Reverse Primer |
| --- | --- | --- | --- |
| GAPDH | Rat | GGGATATGCAAACCTGGGTTTAG | GTTATGGGGTCTGGGATGGAATT |
| OPN | Rat | CACTCCAATCGTCCCTACA | CTTAGACTCACCGCTCTTCAT |
| OCN | Rat | CCTCACTCTTGTCGCCCTG | CTCTTGGACACGAAGGCTGA |
| RUNX2 | Rat | CCTTCCACTCTCAGTAAGAAGA | TAAGTAAAGGTGGCTGGATAGT |
| COL2 | Rat | GACCCCATGCAGTACATG | GACGGTCTTGCCCCACTT |
| SOX9 | Rat | CACACTACAGCCCCTCCTAC | CCTCCTCAAGGTCGAGTGAG |
| ACAN | Rat | TGGTGATGATCTGGCACGAG | CTCCGCTTCTGTAGTCTGCG |
| COL1 | Rat | TGGTGAGACGTGGAAACCTG | CTTGGGTCCCTCGACTCCTA |
| TNMD | Rat | AGAGAACTGTGAGGGCTGTC | GTGCTCCATGCCATAGGTCT |
| SCX | Rat | CTCAGCAACCAGAGAAAGTTGAG | CTTCACTAGTGGCATCACCTCTT |

**References:**

[1]	J. Shi, H. Yao, B. Wang, J. Yang, D. Liu, X. Shang, H. Chong, W. Fei, D. A. Wang, Construction of a Decellularized Multicomponent Extracellular Matrix Interpenetrating Network Scaffold by Gelatin Microporous Hydrogel 3D Cell Culture System, *Macromol Rapid Commun* **2024**, *45* (5), e2300508.

[2]	Z. Wu, H. Yao, H. Sun, Z. Gu, X. Hu, J. Yang, J. Shi, H. Yang, J. Dai, H. Chong, D. A. Wang, L. Lin, W. Zhang, Enhanced hyaline cartilage formation and continuous osteochondral regeneration via 3D-Printed heterogeneous hydrogel with multi-crosslinking inks, *Mater Today Bio* **2024**, *26*, 101080.

[3]	Z. Wu, J. Yang, H. Chong, X. Dai, H. Sun, J. Shi, M. Yuan, D. Liu, M. Dang, H. Yao, W. Fei, 3D-printed biomimetic scaffolds loaded with ADSCs and BMP-2 for enhanced rotator cuff repair, *J Mater Chem B* **2024**, *12* (47), 12365.

[4]	a) W. Song, Z. Ma, X. Wang, Y. Wang, D. Wu, C. Wang, D. He, L. Kong, W. Yu, J. J. Li, H. Li, Y. He, Macroporous Granular Hydrogels Functionalized with Aligned Architecture and Small Extracellular Vesicles Stimulate Osteoporotic Tendon-To-Bone Healing, *Adv Sci (Weinh)* **2023**, *10* (34), e2304090; b) X. Dai, M. Yuan, Y. Yang, M. Dang, J. Yang, J. Shi, D. Liu, M. Li, H. Yao, W. Fei, Dual cross-linked COL1/HAp bionic gradient scaffolds containing human amniotic mesenchymal stem cells promote rotator cuff tendon-bone interface healing, *Biomater Adv* **2024**, *158*, 213799; c) W. Zhao, J. Yang, Y. Kang, K. Hu, M. Jiao, B. Zhao, Y. Jiang, C. Liu, F. Ding, B. Yuan, B. Ma, K. Zhang, A. G. Mikos, X. Zhang, Animal Models of Rotator Cuff Injury and Repair: A Systematic Review, *Tissue Eng Part B Rev* **2022**, *28* (6), 1258.

[5]	C. M. López De Padilla, M. J. Coenen, A. Tovar, R. E. De la Vega, C. H. Evans, S. A. Müller, Picrosirius Red Staining: Revisiting Its Application to the Qualitative and Quantitative Assessment of Collagen Type I and Type III in Tendon, *J Histochem Cytochem* **2021**, *69* (10), 633.

[6]	a) Z. Luo, F. Wu, E. Xue, L. Huang, P. Yan, X. Pan, Y. Zhou, Hypoxia preconditioning promotes bone marrow mesenchymal stem cells survival by inducing HIF-1α in injured neuronal cells derived exosomes culture system, *Cell Death Dis* **2019**, *10* (2), 134; b) R. Saleem, S. Mohamed-Ahmed, R. Elnour, E. Berggreen, K. Mustafa, N. Al-Sharabi, Conditioned Medium from Bone Marrow Mesenchymal Stem Cells Restored Oxidative Stress-Related Impaired Osteogenic Differentiation, *Int J Mol Sci* **2021**, *22* (24).
